# Physical modeling of the effect of shape, blockage, and flow variability on scour in culvert outlets

**DOI:** 10.1371/journal.pone.0306252

**Published:** 2024-06-27

**Authors:** Kaywan Othman Ahmed, Mohammad Reza Kavianpour, Ata Amini, Younes Aminpour

**Affiliations:** 1 Department of Civil Engineering, K. N. Toosi University of Technology, Tehran, Iran; 2 Faculty of Engineering, Department of Civil Engineering, Tishk International University—Sulaimani, Kurdistan Region, Iraq; 3 Department of Civil Engineering, K.N. Toosi University of Technology, Tehran, Iran; 4 Kurdistan Agricultural and Natural Resources Research and Education Center, AREEO, Sanandaj, Iran; 5 Department of Hydraulic, Hydro-Environmental Engineering, Water Research Institute, Ministry of Energy, Tehran, Iran; National Sun Yat-sen University, TAIWAN

## Abstract

The widespread use of culverts has prompted researchers to focus on developing precise designs to prevent their failure caused by scouring at the culvert outlet. This study employed physical modelling to investigate alternation in culvert outlets under different conditions, including variations in culvert shape, blockage, and flow discharge during steady and unsteady flow conditions. Box and circular culverts were examined with 0%, 15%, and 30% blockage rates at the culvert inlet. For unsteady flow conditions, two hydrographs were generated, each with nine distinct flow discharges, while for steady flow conditions, flow rates of up to 14 *l/s* and 22 *l/s* were used. The sediment and flow conditions were carefully selected to ensure clear water throughout the experiments. According to the study results, the scour profile exhibited more growth in the circular culvert compared to the box culvert across all cases. Furthermore, an increase in flow rate led to an increase in the scour hole dimension, and the scouring increased with a rise in hydrograph stepwise. However, when the degree of blockage was increased, a strictly proportional increase in scour depth was not observed across all cases. The results and data presented in this research can be used by other researchers in addition to being used by hydraulic designers.

## Introduction

Culverts, commonly used in engineering to manage stormwater flow through roadways, undergo thorough design and hydraulic calculations to prevent issues. However, poorly designed culverts can be prone to erosion at both ends, leading to destabilization and collapse. This collapse can cause significant damage to nearby structures, requiring costly reconstruction and flood-related repairs. Culvert design involves determining optimal dimensions for hydraulic capacity and incorporating protective measures outlet to prevent erosion-related damage [1]. Numerous variables contribute to the scour phenomenon occurring downstream of a culvert outlet. The evaluation of the hydraulic performance of culverts remains challenging due to the presence of numerous types of floating debris, regardless of the simplicity of their structural design [2,3].

The culvert shape and inlet blockage are prominent characteristics that significantly influence the scouring process. In their investigation into how culvert shape affects scour depth at the culvert’s outlet, [4] observed that the scour dimensions for circular culverts differ considerably from those of other culvert types. [5] investigated the scouring at rectangular, square, circular, and arch culverts and found that the culvert shape has limited effects on outlet scour. Contrarily, the findings of [6] showed that the dimensions of scouring at the outlet of culverts with circular shapes differ significantly from those observed in culverts with alternative shapes. [7] conducted a study wherein they noticed that square culverts exhibited more excellent dimensions in terms of scour hole length and width when compared to circular culverts. Nevertheless, square culverts exhibited a slightly reduced maximum scour depth within the scour hole.

Moreover, the blockage of culverts not only risks private properties but also poses a threat to public assets, amplifying the potential for consequential damage. The initial category typically comprises studies grounded in field data collected post major flood events. Within these investigations, the emphasis lies on identifying the factors contributing to blockage and understanding the repercussions of such obstructions on downstream flow paths. As an illustration, [8] gathered field data following a flood event, revealing a heightened risk of culvert blockage when the opening size is less than 6 m (measured diagonally). Additionally, [9] provided estimates of culvert and bridge blockage, considering factors such as the availability, mobility, and transportability of debris. Also, they delved into the mechanisms of culvert blockage and its influence on flood dynamics. Numerous studies have investigated culvert blockage and impacting on downstream socur. For instance, [10] reported 22% increase in scour depth, 25% increase in scour width and up to 60% increase in scoured area along scour hole centerline. [11] reported that when a culvert is partially blocked, the location of maximum scour depth occurs in a closer distance from the outlet. Therefore, they compared their work with some of the recent studies and concluded that the blockage at the culvert inlet can be one of the influencing factors in estimating the scour depth.

As outlined by [11], their findings in partially blocked conditions revealed a distinct scouring bed profile. Specifically, a significant proportion, ranging from 88% to 98%, of the maximum scour depth occurred during the rising limb of the hydrograph under unsteady flow conditions. In cases of partial blockage, both the maximum scour depth and the scoured area demonstrated larger dimensions. Notably, during the rising limb of the hydrograph, the maximum scour depth was observed to be farther from the culvert outlet. [12] directed their attention to steady flow conditions, aiming to address the existing gap by examining scouring at the outlet of both partially and non-blocked culverts. Their investigation revealed that, when compared to equivalent non-blocked culvert conditions, both the scoured area and maximum scour depth experienced an increase. Specifically, the scoured area at blocked culverts was found to be 20–60% larger in comparison to non-blocked cases. Furthermore, under partially blocked conditions, the scouring width and length increased by up to 17% when contrasted with non-blocked scenarios. [13] found that the impact extended beyond the maximum scour depth, influencing both the scour area and sediment volume. Notably, there was a substantial increase in near-wall scouring, and the scour hole extended along the flow direction due to the accumulation of debris. The study underscored distinct differences in scour parameters between cases with blockage and those without. examined the repercussions of inlet blockage on culvert efficiency and scour depth. Their findings revealed that with an incremental increase in blockage ratio by 10%, 20%, and 30%, the relative scour depth similarly increased by 2.63%, 5.78%, and 10.53%, respectively, when compared to non-blocked cases.

In previous studies, scientists mainly looked at factors affecting the scouring in the downstream of culverts. However, there’s a lack of information on partially blocked conditions for box and circular culverts. This study utilized physical modeling to investigate scour alterations at culvert outlets, considering diverse conditions such as variations in culvert shape, blockage, and flow discharge under both steady and unsteady flow conditions. One of the main goals of the current research is to investigate the steady and unsteady flow conditions and the process of changes in the hydrograph entering the culvert on the changes in the scour hole, which has yet to be studied in previous studies. Also, investigating the dimensions and geometry of the scour hole for two different shapes of Calvert (box and circle) is another goal of this research. The other innovations of this research include analyzing and evaluating different values of culvert blockage and its influence on the dimensions of the scour hole in steady and unsteady flow conditions. We examined box and circle culverts with blockage rates set at 0%, 15%, and 30% at the culvert inlet. Unsteady flow conditions involved two hydrographs, each with nine distinct flow discharges, while steady flow conditions included rates of up to 14 *l/s* and 22 *l/s*. The data and analysis presented in this research provide useful and practical information for researchers and hydraulic engineers to design and prevent scouring at the culvert outlet under different flow conditions and the shape and blockage of the culvert.

## Material and methods

### Experimental setup

**Fig 1:**
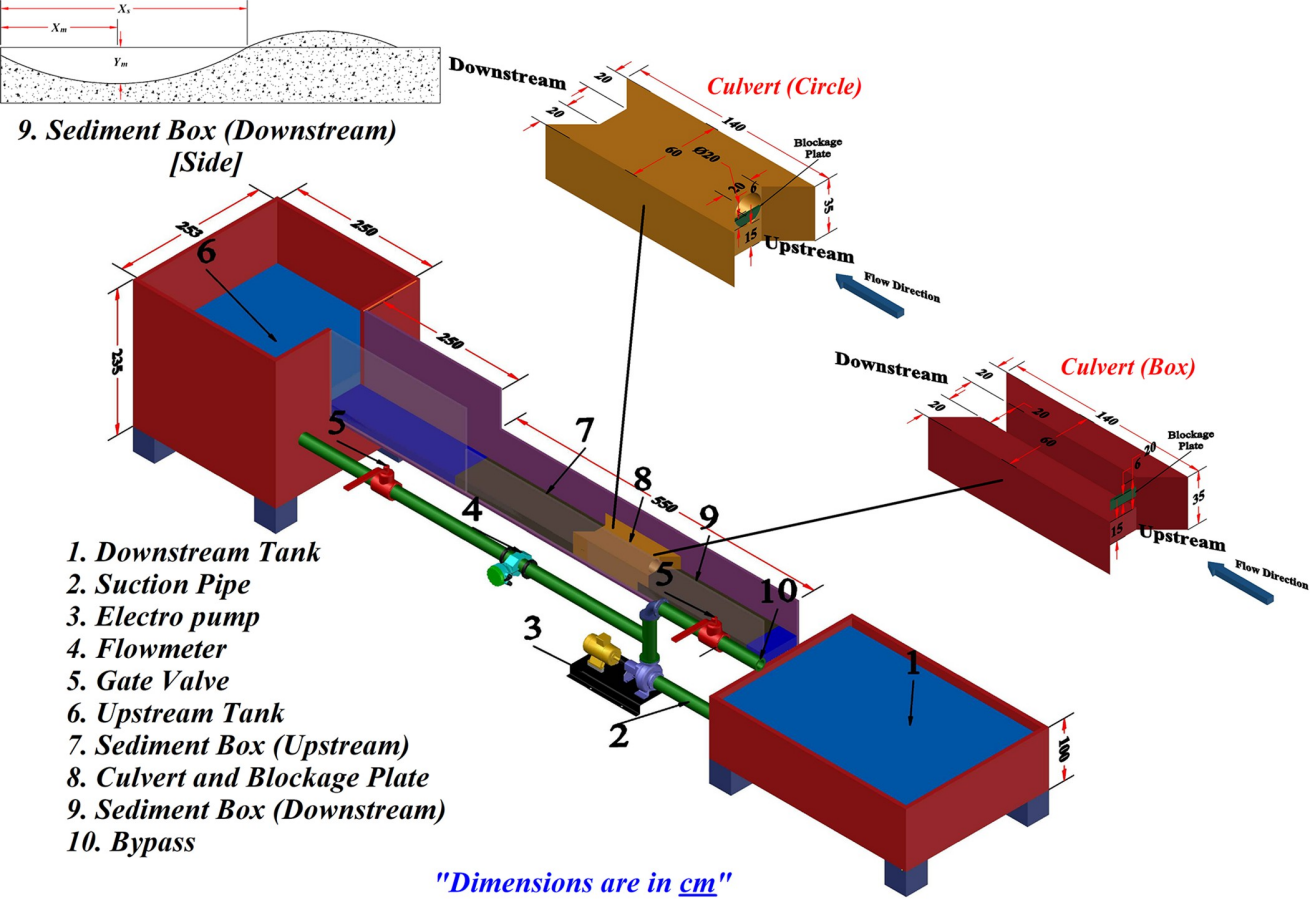
Configuration of flume and culvert model.

The present study involves the utilization of experimental data to determine scour at the culvert outlet. Experiments were carried out at the Hydraulics Laboratory of the University of Sulaimani (UoS), Kurdistan, Iraq. A Perspex flume with a length of 7.9 m, a depth of 0.7 m, and a width of 0.6 m was used. The flume exhibited a consistent inclination of 0.001 in conjunction with a water recirculation mechanism. The culvert inlet was positioned at a distance of 4.6 m from the flume inlet to ensure that the flow reaching the test area was fully developed. The experimental setup comprised of a sand basin with a length of 4.4 m and a depth of 0.15 m, which had a width equivalent to that of the flume. The median grain size of sands was found to be *d*_*50*_ = 1.1 mm, with a geometric standard deviation of *σ*_*g*_ = 2.9 mm. This range falls within the category of uniform sediment [14,15]. The sediment was graded to conform to the elevation of the culvert inlet and outlet. Fig 1, depicts the specifics of the flume with culvert model.

The values of critical shear velocity (, $u_{{*}_{c}}$) and critical flow velocity (*V*_*c*_) were presented based on the equations proposed by [14]. These equations are as follows:

$$u_{{*}_{c}}=0.0305d_{50}^{0.5}+0.0065d_{50}^{-1}$$ (1)

$$\frac{V_{c}}{u_{{*}_{c}}}=5.75\;\log[5.53\frac{y_{w}}{d_{50}}]$$ (2)

where *y_w_* is the water depth. The critical shear velocity and critical flow velocity values were obtained as $u_{{*}_{c}}$ = 0.314 m/s and *V*_*c*_ = 0.425 m/s, respectively. All the tests were done in clear water conditions.

In order to attain optimal water clarity for experimental purposes, the flow intensity was deliberately chosen to be below the threshold required for sediment entrainment. The flow depth was measured at upstream and downstream of the culvert using a point gauge with an accuracy of ±0.1 mm. Each experimental condition was conducted under unblocked and partially blocked with a 15% and 30% blockage rate.

This research gathered with Reynolds numbers Re > 10^5^ to mitigate scale effects on scour depth, following [16]. For Re > 10^4^, viscous effects become negligible [17]. The flume width, a key factor in scour studies for obstacles in flow, and its effects on scouring were considered [18]. The culvert width to flume width ratio was maintained at approximately 1:3 for the data in this study, potentially influencing scour dimensions and mechanisms. To minimize the effects of constriction on the dimensions of the scour hole, the geometry of the culvert remained constant in both box and circular types. In other words, in all tests, the cross-section of the inlet flow for the box culvert was 0.2 x 0.2 m^2^, and for the circular culvert, the diameter was considered to be 0.2 m.

### The experiments

In this research, two culverts were investigated with Circle and Box shapes. The length of the culvert in both cases was 1 m. The dimensions of the inlet and outlet sections of the culvert were 0.2m x 0.2 m for the box culvert, and the diameter was 0.2 m for the circle culvert. To reduce the turbulence and ensure the flow’s full development, 30° flare transitions were used in the inlet and outlet sections of the culvert [19]. Two symmetrical hydrographs were investigated for unsteady flow in the range of 6–22 *l/s* (first hydrograph) and 2–14 *l/s* (second hydrograph). Each time step from the first hydrograph was 40 minutes. It took 25 minutes for the second hydrograph. Also, the scour hole development process in steady flow conditions was measured in a fixed time for the maximum flow rate in each hydrograph (22 *l/s* for the first hydrograph and 14 *l/s* for the second hydrograph). In partial blockage, the submerged upstream and downstream of the culvert can cause a transition from supercritical to subcritical flow within the culvert, occurring near its terminus. The capacity of the culvert determined the hydrograph’s maximum flow. Table 1 presents the details of the experiments conducted in this research.

**Table 1:** Experimental cases.

Models	Flow Conditions	Maximum Discharge *Q*_*M*_ (*l/s*)	Blockage (%)	Time (min)
Box	Unsteady	22	0	360
			15	360
			30	360
		14	0	225
			15	225
			30	225
	Steady	22	0	360
			15	360
			30	360
		14	0	225
			15	225
			30	225
Circle	Unsteady	22	0	360
			15	360
			30	360
		14	0	225
			15	225
			30	225
	Steady	22	0	360
			15	360
			30	360
		14	0	225
			15	225
			30	225

### Dimensional analysis

Scouring at the outlet of culverts can generally be represented as a functional relationship of several variables, which can be expressed as Eq 3 [20]:

$$f_{1}(W,S_{0},D,F,T,\rho ,\rho_{w},V,V_{w},\mu ,g,H_{e},H_{u},L_{w},h_{c},L_{c},X,A_{w},A_{c})=0$$ (3)

where *W* is the width of the upstream channel, *S*_*0*_ is the bed slope, *D* is the diameter of wooden debris, *F* is the feeding rate of debris into the flow, *T* is the time after feeding from the falling of the first debris, *ρ* is the fluid density, *ρ*_*w*_ is the wooden debris density, *V* is the average flow velocity at upstream, *V*_*w*_ is the velocity of woody debris, *μ* is the dynamic viscosity of water, *g* is the acceleration due to gravity, *H*_*e*_ is the flow depth at the culvert inlet, *H*_*u*_ is the upstream flow depth, *L*_*w*_ is the length of debris, *h*_*c*_ is the culvert width or diameter for the box and pipe culverts, respectively, *L*_*c*_ is the culvert length, *X* is the distance between the accumulated debris and the channel bed at the inlet, *A*_*w*_ is the area of the culvert opening occupied by the debris, and *A*_*c*_ is the total area of the culvert opening. By applying Buckingham’s *Pi* theory, Eq 3 can be regrouped as Eq 4:

$$B,X*=f_{2}(Q*,T*,Re_{w},F_{ru},F_{re},\rho *,h_{c}*)$$ (4)

in which *B* = *A*_*w*_/*A*_*c*_ is called the degree of culvert blockage, X* = *X*/*H*_*e*_ is called the non-dimensional blockage height, T* = *TV*/*H*_*e*_, Q* = *VH*_*e*_^*2*^/*F*, ρ* = *ρ*_*w*_/*ρ*, *h*_c_* = *H*_*e*_/*h*_*c*_, *Re*_*w*_ = *ρ*_*w*_*V*_*w*_*D*/*μ* is the Reynolds number of debris, $F_{re}=V/\sqrt{gH_{e}}$ ∙is the Froude number at the culvert entrance, and $F_{ru}=V/\sqrt{gH_{u}}$ is the upstream flow Froude number. The culverts are generally under inlet control conditions during floods and the process is influenced by the upstream flow characteristics. Therefore, in the present study the upstream Froude number has been used.

This study opted for the rate of blockage (*B*), as defined by [11], instead of the traditionally used hydraulic blockage parameter [21], based on the percentage of the culvert opening blocked by debris. It is important to highlight that certain constants in this study, including the channel bed slope, nondimensional channel width, upstream flow Reynolds number, nondimensional debris length, and nondimensional culvert length, were omitted from Eq (4).

## Result and discussion

### Unsteady flow conditions

To illustrate the effects of discharge on scour, we used two distinct hydrographs for both circular and box culverts. This section presents the results of tests with a hydrograph with *Q*_*M*_ = 22 *l/s* as the first hydrograph and *Q*_*M*_ = 14 *l/s* as the second hydrograph, where *M* stands for maximum.

#### The first hydrograph

**Fig 2:**
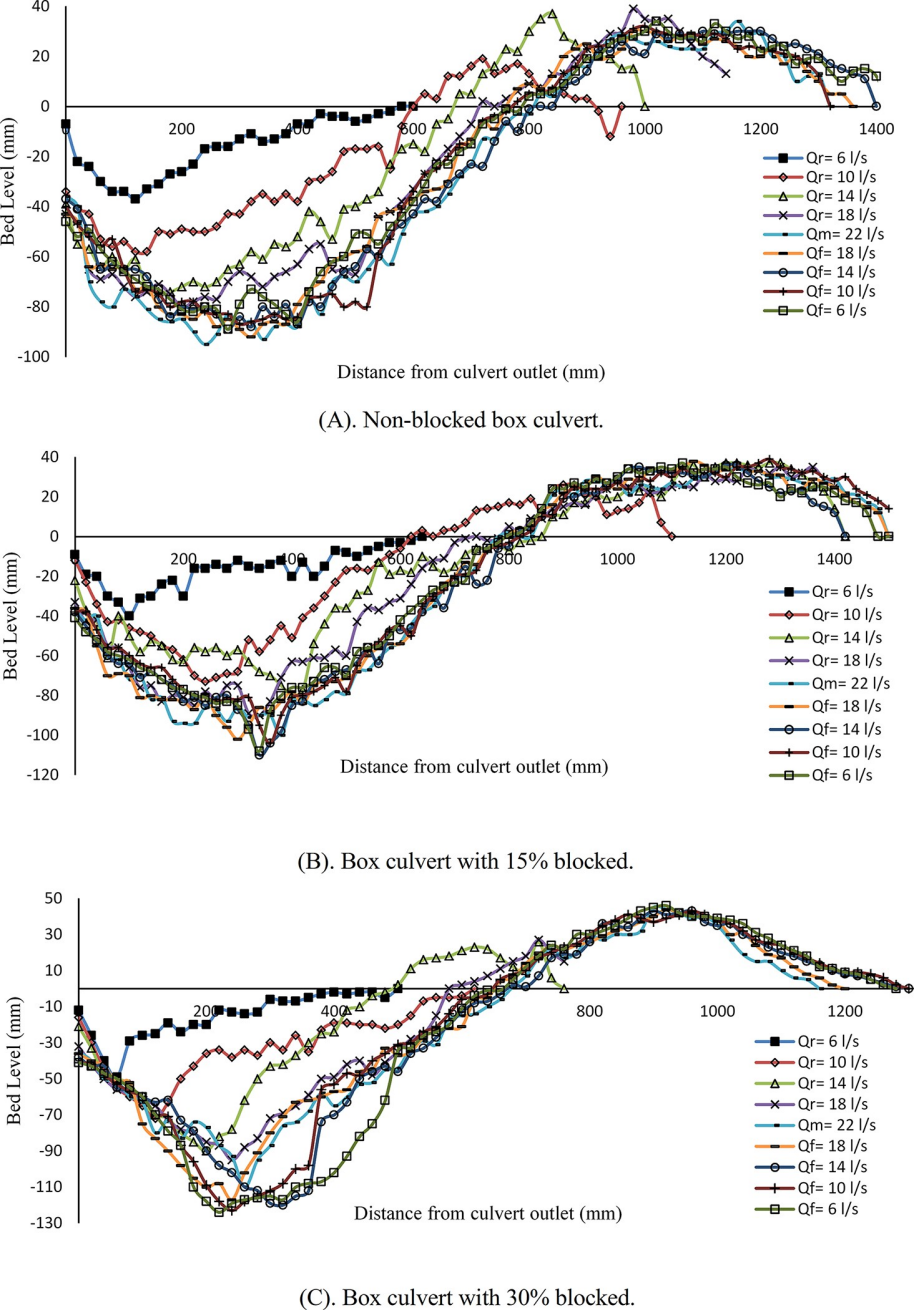
Scour profile for box culvert. (A) non-blocked, (B) culvert with 15% blocked, and (C) culvert with 30% blocked. (*r*, *m*, and *f* stand for raising limb, maximum flow rate, and falling limb of hydrograph, respectively).

The scouring profile formation in the box downstream of non-blocked and blocked culverts with different flow rates under unsteady flow conditions is illustrated in Fig 2. Fig 2 shows that the minimum flow rate started at 6 *l/s* and raised to a maximum of 22 *l/s* and then fell to 6 *l/s*. In Fig 2(A), the flow rate was little sufficient to move the sediment in the first stepwise, and some motion in the sediment was observed as the flow rate increased to *Q*_*r*_ = 10 *l/s* and *Q*_*r*_ = 14 *l/s*, where *r* stands the rising limb of the hydrograph. The maximum scouring depth (*d*_*sm*_) proportion increased when the flow rate reached its maximum (*Q*_*M*_ = 22 *l/s*) and was about 0.095 m. The scouring depth increased and approximately remaining constant for other stepwise (*Q*_*r*_). As shown in Fig 2(B), with 15% inlet blockage of the box culvert, the *d*_*sm*_ in the first step of the hydrograph was about 0.04 m. As the discharge increases, the scour depth and sediment deposition increase. The maximum scour depth in the last hydrograph step, *Q*_*f*_ = 6 *l/s*, was 0.108 m. Fig 2(C) illustrates the scour variation in the 30% inlet block of the box culvert against discharge. The scouring hole forms close to the outlet of the culvert. The scouring depth increased by increasing the flow rate in each stepwise. In addition, when the discharge decreased, the maximum rate of scouring increased, and in the last step of the hydrograph, it reached 0.124 m. Box culvert studies show a direct correlation between blockage rate and maximum scour depths. A 15% blockage led to a 13.7% increase in scour depths. A 30% blockage resulted in a roughly 23.4% increase compared to the non-blocked condition, indicating a significant acceleration in the scouring process. The result obtained was the same as the achievements [3,11,22].

**Fig 3:**
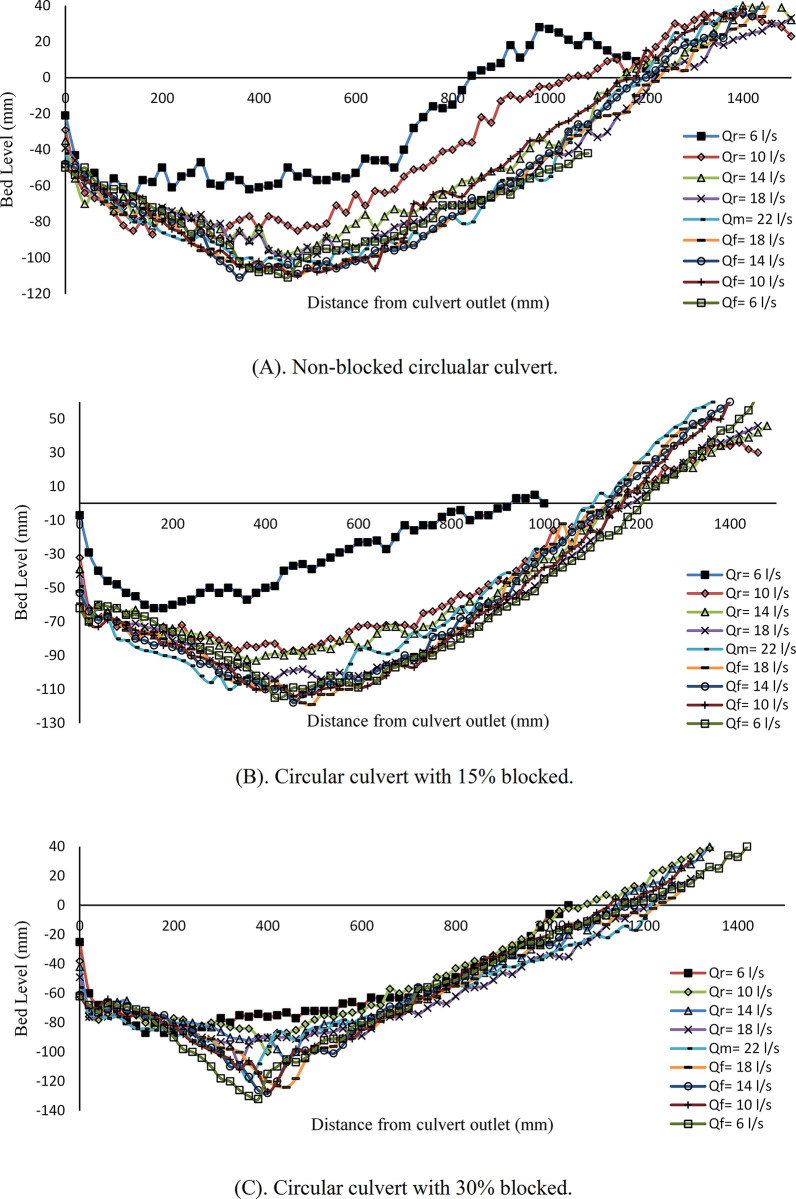
Scour profile of circular culvert. (A) non-blocked; (B) with 15% blocked, and (C) with 30% blocked.

Fig 3 shows the scouring profile of a circular culvert with varying flow rates. Fig 3(A) illustrates the fully opened culvert case, and the noticeable scouring happened with the first stepwise hydrograph as *Q*_*r*_ = 6 *l/s*. The maximum scour reaches approximately 0.066 m, which reveals that the rate of change to the box culvert increased by approximately 44%. The scouring depth was observed to grow as the discharge rate was incrementally increased in every stage. The highest scour depth occurred when the flow rate declined gradually to *Q*_*f*_ = 14 *l/s*, resulting in a depth of approximately 0.111 m. This depth remained constant for the remaining falling limb steps. Therefore, the maximum scour depth increased by 14.4% compared to a non-blocked box culvert. Fig 3(B) shows the scouring variation with 15% inlet blockage. At the initial discharge rate of *Q*_*r*_ = 6 *l/s*, the observed maximum scouring depth reached 0.062 m. The rate of scour hole deformation exhibits a significant increase over subsequent stages of the hydrograph compared to the first stepwise. The maximum scour depth recorded in the sixth step of the hydrograph, with the flow rate of *Q*_*f*_ = 18 *l/s*, is about 0.119 m. This amount decreased for reaming falling limb steps.

The data indicates a rate of change of about 6.7% when comparing the non-blocked circular culvert to the partially 15% blocked circular culvert. Fig 3(C) shows the scour profile with a 30% inlet blockage rate. In this case, more scour happened in the outlet of the culvert compared to previous cases. The scour hole increased proportionally with the change in flow rate. The maximum scour depth recorded in the final step of the hydrograph with *Q*_*f*_ = 6 *l/s* was 0.132 m. A 30% obstruction results in a significant increase of 9.8% and 15.9% in the maximum scour depth, compared to 15% inlet blockage and no blockage, respectively. Overall, the comparison between the box and the circular culvert indicates that the scour profile exhibited more growth in the circular culvert than the box culvert across all cases. Therefore, the shape of a culvert is among the factors that impact the scouring process, as stated by [5].

#### The second hydrograph

**Fig 4:**
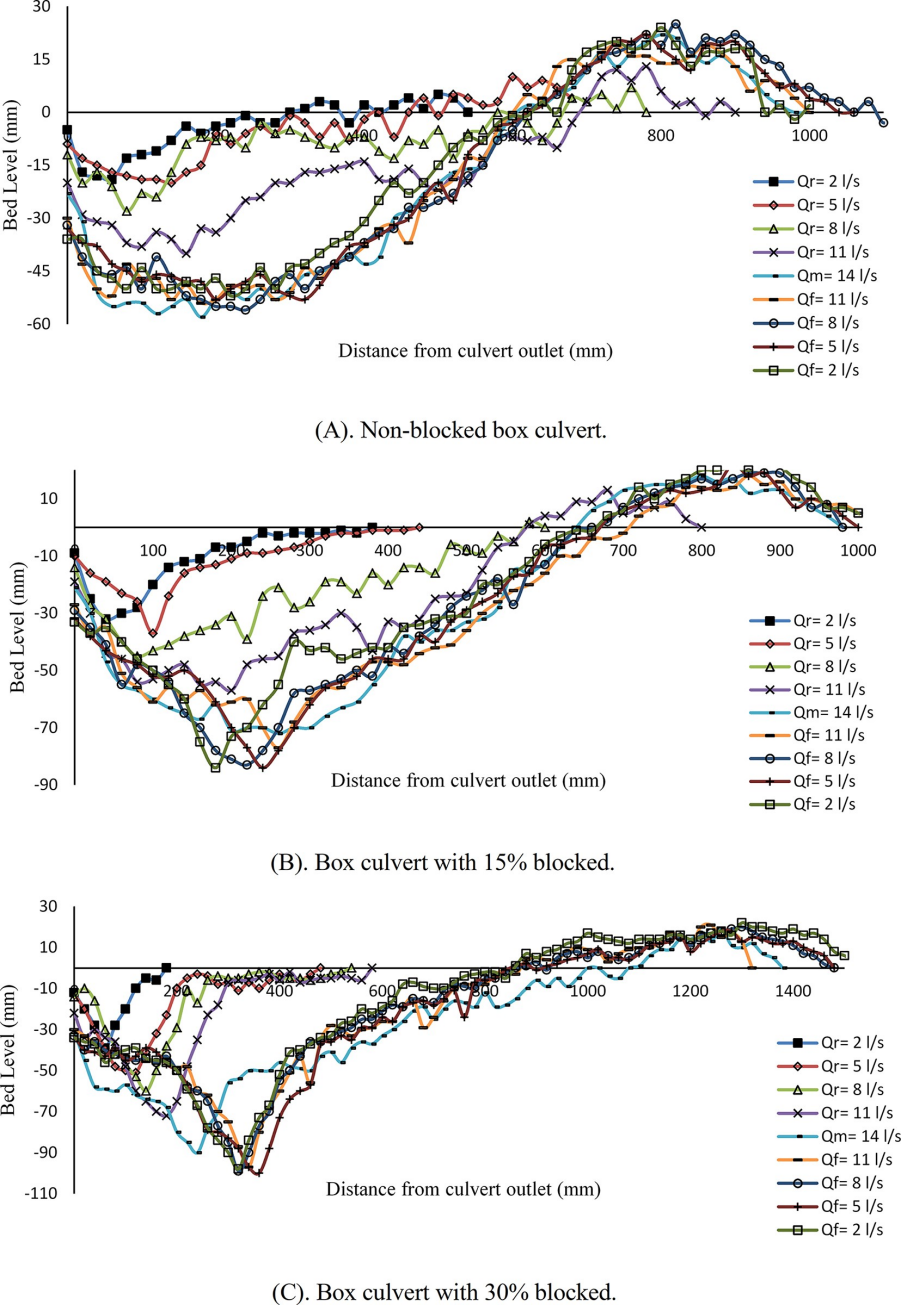
Scour profile for box culvert. (A) non-blocked, (B) with 15% blocked and (C) with 30% blocked.

Fig 4 presents the variation of the scour profile at the box culvert under unsteady flow conditions with a *Q*_*M*_ = 14 *l/s*. The analysis considers experiments with nonblocked and blocked cases. The second hydrograph has a symmetrical pattern, commencing at a flow rate of *Q*_*r*_ = 2 *l/s*, reaching a peak rate of *Q*_*M*_ = 14 *l/s*, and declining to *Q*_*f*_ = 2 *l/s*. Fig 4(A) depicts the scouring profile for a nonblocked culvert, revealing a notable rise in scouring throughout the initial three steps. The scouring further intensified when the flow rate reached *Q*_*r*_ = 11 *l/s*. The scouring hole exhibited a notable increase throughout the subsequent incremental steps of the hydrograph. The maximum scour depth of 0.058 m was recorded with a maximum flow rate *Q*_*M*_ = 14 *l/s*.

Fig 4(B) illustrates the impact of 15% inlet blockage of box culvert. The scouring notice in proximity to the culvert outlet for the initial flow rates of 2 *l/s* and 5 *l/s*. As the discharge rate increased, the size of the scouring hole grew in direct proportion. The findings indicate that a 15% blockage resulted in a 31% increase in the maximum scour depth compared to the blockage. Fig 4(C) illustrates the impact of a 30% obstruction on the scouring profile. The initial four steps of the hydrograph indicate that scouring occurred near the culvert outlet.

In contrast, the scouring process occurred considerably from the outlet throughout the remaining steps. The observed maximum scour depth was 0.1 m in the falling limb under a flow rate of *Q*_*f*_ = 5 *l/s*. The findings indicate that the presence of a 30% blockage has a considerably more significant influence than the 15% blockage and no blockage, so the scour increased by 16% and 42%, respectively.

**Fig 5:**
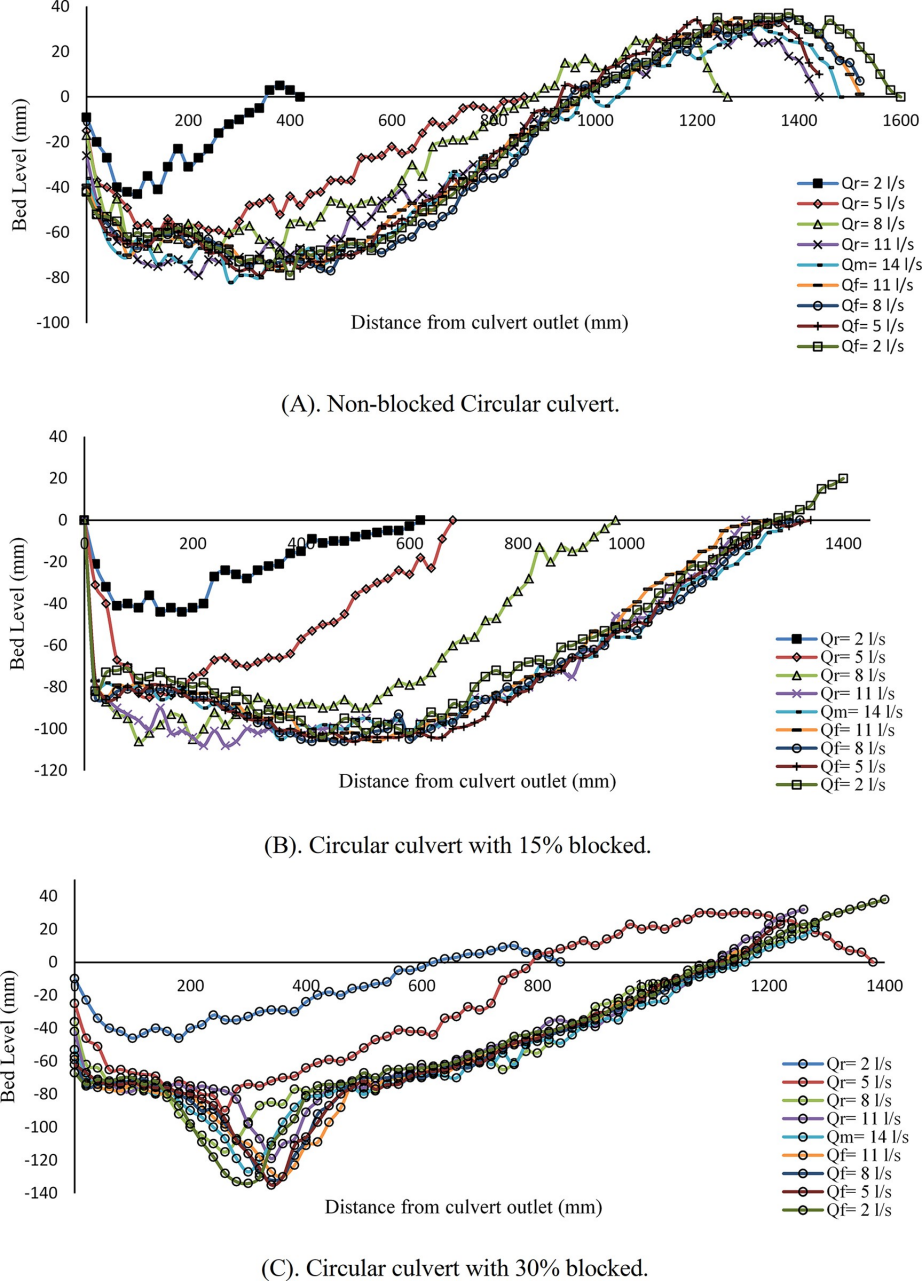
Scour profile for circular culvert. (A) non-blocked, (B) with 15% blocked and (C) with 30% blocked.

The scouring profile of a circular culvert for the second hydrograph is illustrated in Fig 5. The scour profile for a fully opened culvert is depicted in Fig 5(A). Compared to other stepwise hydrographs, the first flow rate of *Q*_*r*_ = 2 *l/s* affects scouring and creates a small hole with a maximum scour depth of 0.043 m. Raising the flow rate increased sediment motion and moved away from the culvert outlet. The maximum scour depth was recorded at a maximum flow rate of *Q*_*M*_ = 14 *l/s*, approximately 0.08 m. Fig 5(B) shows scouring at the culvert with 15% blockage. The scouring hole directly correlates with the flow rate, as it consistently enlarges with an increase in the flow rate. The maximum scour depth occurred when the flow rate reached *Q*_*f*_ = 8 *l/s*, and the corresponding depth was 0.106 m. Hence, the data demonstrates a 24.5% rise in the maximum scour depth when comparing the 15% blocked inlet case to the 0% blocked case. Fig 5(C) illustrates the scouring profile with a 30% inlet blockage. The sediment deformation was observed when the initial flow was *Q*_*r*_ = 2 *l/s*; maximum scour depth resulted in a sediment movement of 0.046 m. However, when the discharge was increased to *Q*_*r*_ = 5 *l/s*, the scouring hole deepened by 0.09 m. This shift was evident when compared to subsequent incremental increases in discharge. The expansion of the scour hole is seen to occur at a distance ranging from 0.25 m to 0.35 m downstream of the culvert. A measurement of 0.132 m was obtained from the maximum scour depth when the flow rate reached *Q*_*f*_ = 5 *l/s*. The findings indicate that the presence of a 30% blockage with a second hydrograph significantly affects the formation of scouring. Additionally, the rate of change increases in scouring and was observed to be 21% and 41% for partially 15% blocked and nonblocked cases, respectively.

The scouring processes in the box and circular culverts were compared using the flow rate variation in both hydrographs with varying blockage rates. The findings show that the scouring depth increased with increased hydrograph stepwise and blockage rate. When comparing the same rate of blockage and non-blockage, it was found that the circular culvert indicated more scouring depth than the box culvert overall. These findings are consistent with [11].

### Steady flow conditions

**Fig 6:**
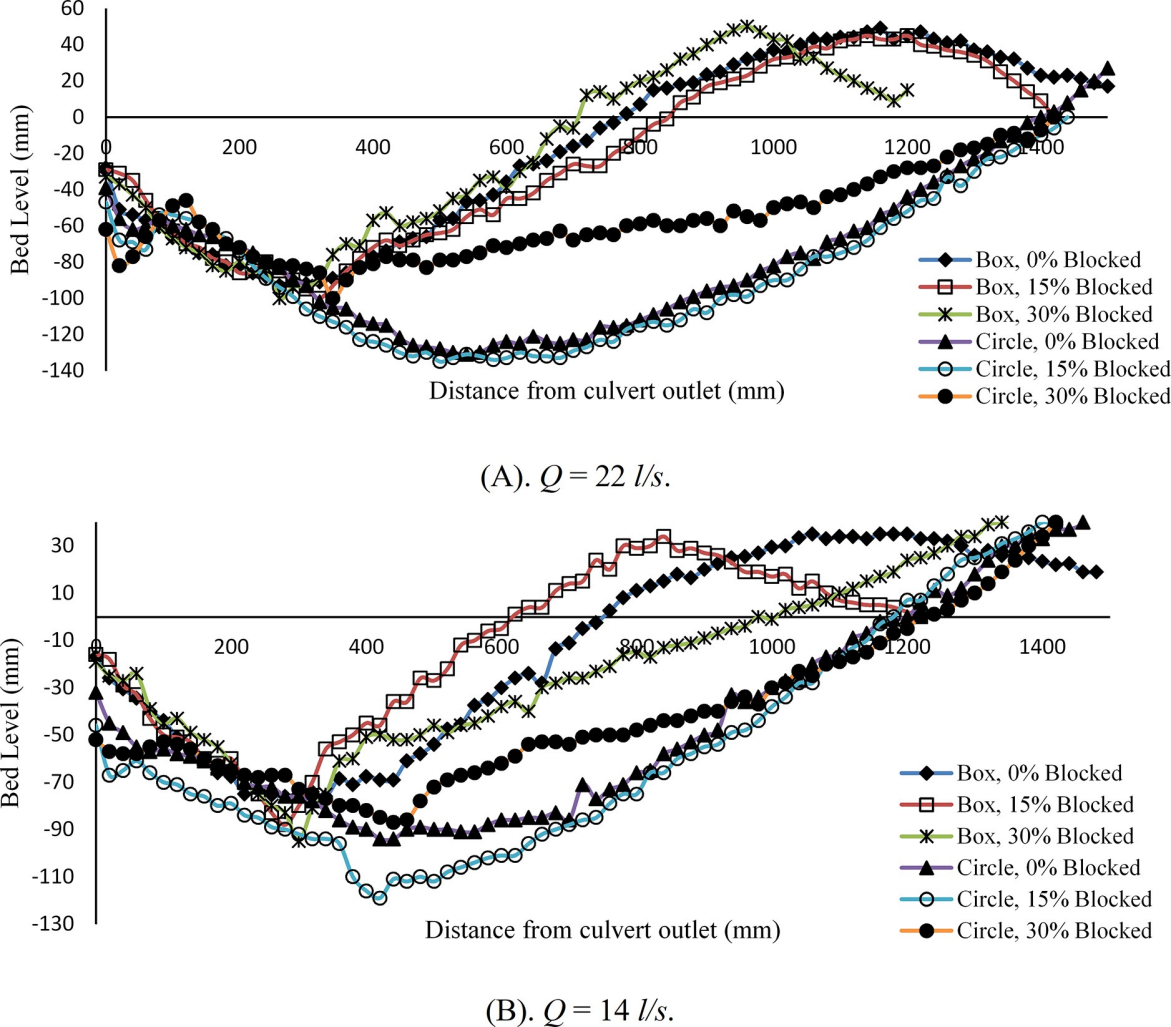
Scour profile for box and circular culvert with different blockage rates. (A) *Q* = 22 *l/s*, *t* = 360 min and (B) *Q* = 14 *l/s*, *t* = 225 min.

Fig 6 illustrates a comparative analysis of scour profile development in downstream locations under steady flow conditions for box and circular culverts. Fig 6(A) shows the scour profile for *Q*_*M*_ = 22 *l/s* under 0%, 15%, and 30% inlet blockage for *t* = 360 min duration. When observing the sediment movement within the box culvert, it becomes evident that under 0% blockage, the maximum depth of scour reaches 0.093 m. However, with a 15% inlet blockage, it increases to 0.10 m, and with a 30% blockage, the maximum depth of scour is recorded as 0.094 m. An analysis of these data highlights that the 15% blockage results in the highest *d*_*sm*_, followed by 30% blockage condition, and then 0% blocked case. Consequently, it is evident that, in this particular case, an increase in the rate of blockage does not lead to a proportional increase in scour depth. Furthermore, it’s notable that the 15% blockage configuration creates the largest scour hole along the downstream length, followed by the unblocked case, and lastly, the 30% blockage case. In the condition of 15% blockage, the flow comes out from under the blockage plate with a shallow depth, and its flow regime is supercritical (Fr>1). In this case, the velocity of the flow is high, and the supercritical flow tends to turn into a subcritical flow, and this transformation will be done through the hydraulic jump. Because the velocity of the outflow from in the blockage is 15% higher than the velocity of the flow in the blockage 30%, the progress of the hydraulic jump in the blockage is 15% towards the downstream. The flow is more turbulent, so the power of destruction is Higher, and it reaches the scour hole. This is why, in 15% blockage, the scour hole with larger dimensions is formed compared to 30% blockage.

On the other hand, the tailwater depth always acts as a cushion against the flow and by dissipating the flow’s energy, it tries to reduce the flow’s destructive power and helps to reduce the dimensions of the scour hole. In 15% blockage, the tailwater depth is less than in 30% blockage. This issue is also one of the reasons for the larger dimensions of the scour hole in 15% blockage compared to 30% blockage.

Another issue that can be the reason for the larger dimensions of the scour hole in the 15% blockage compared to the 30% blockage is the flow passing over the blockage plate. In 15% blockage, because the blockage plate is at a lower level, a part of the upstream flow tends to pass over the plate simultaneously as it passes under the plate. This issue will increase the turbulence of the flow and the destructive power of the output flow, and consequently, the dimensions of the scour hole will increase.

In the case of the circular culvert, under the same flow rate of *Q*_*M*_ = 22 *l/s*, the depth of scour measurements varies. In the unblocked case, the *d*_*sm*_ is approximately 0.131 m, followed by a 15% blockage case around 0.135 m, and finally, a 30% blockage results in approximately 0.1 m. Once again, these observations affirm that an increase in the rate of inlet blockage does not necessarily lead to a higher degree of scouring. In this context, the 15% blockage configuration yields the greatest scour depth, followed by the unblocked condition, and lastly, the 30% blockage case. Furthermore, to classify the scour hole along the downstream length, the same cases of box culvert repeated for the circular case. Fig 6(B), shows the scour development for a second flow rate of *Q*_*M*_ = 14 *l/s* under varying rates of blockage with a duration of *t* = 225 min. It is evident that sediment movement is noticeable in all cases. When there is 0% inlet blockage at the culvert, the *d*_*sm*_ records a scour depth of 0.076 m. However, with a 15% blockage, the scour depth increases to around 0.088 m, and with a 30% blockage, it reaches 0.095 m. The findings suggest that an increase in the blockage rate does not have a strictly proportional effect on the formation of scour holes downstream. Unpredictably, the culvert with 15% blockage results in the most significant scouring hole, followed by the non-blocked case, and then the 30% blocked case.

For the circular culvert under *Q*_*M*_ = 14 *l/s* and various degrees of blockage, our study revealed the following *d*_*sm*_ measurements. In the absence of blockage, the *d*_*sm*_ recorded a scour depth of approximately 0.094 m. However, with a 15% blockage, this depth increased to 0.119 m, and with a 30% blockage, it decreased to 0.087 m. The *d*_*sm*_ values for different blockage cases yielded roughly similar scouring depths along the downstream of the culvert. Therefore, it is evident that a 15% blockage leads to the formation of the most significant scour hole, followed by the non-blocked case, and then the 30% blockage. Furthermore, our findings highlight that an increase in the rate of blockage does not result in a strictly proportional increase in scour depth.

In summary, this study demonstrates notable trends in the context of steady flow conditions. Whenever, the flow rate changed from 14 *l/s* to 22 *l/s* in cases, an increase in the scour depth along the downstream section of the culvert was observed. Conversely, when the degree of blockage increased, it was not observe a strictly proportional increase in scour depth across all cases. Instead, variations were observed, with some cases showing increased scour depths. This behavior can be attributed to the phenomenon of water overflowing the blockage plate, especially when the degree of blockage is higher. Consequently, the water velocity in these cases decreased compared to others, influencing the scouring process. In the case of 15% blockage, the results are consistent with [11] however, the trend is not same as that reported by [7].

### Maximum scour depth

**Fig 7:**
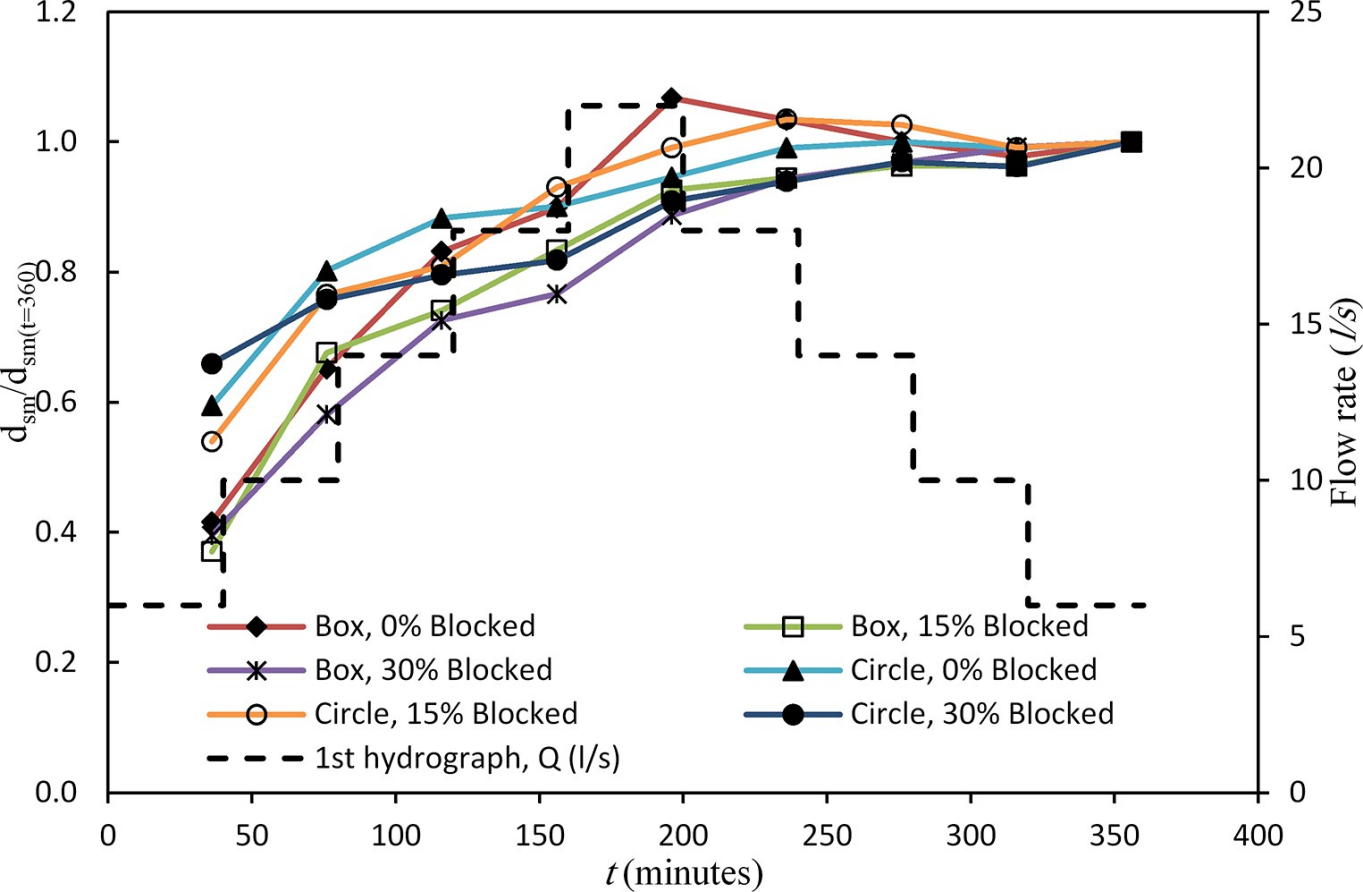
Comparison of *d*_*sm*_ between box and circular culvert first hydrograph (*Q*_*M*_ = 22 *l/s*).

The depth and location of the scour at the outlet of a culvert play a pivotal role in determining culvert design and safety [12]. Fig 7 presents the maximum scour depth in box and circular culvert shapes under unsteady flow conditions with varying blockage rates. The *d*_*s*_/*d*_*sm*_ ratio was employed to normalise the maximum scour depth, where *d*_*s*_ represents the scour depth, and *d*_*sm*_ is the maximum recorded scour depth. The *d*_*sm*_ occurs at *t* = 360 min, with an initial flow rate ranging from *Q*_*r*_ = 6 *l/s* to *Q*_*M*_ = 22 *l/s* and then falling the limb to *Q*_*f*_ = 6 *l/s* for the first hydrograph.

In Fig 7, for the box culvert, during the rising limb (*Q*_*r*_) of the hydrograph, with flow rates of 6, 10, 14, 18, and *Q*_*M*_ = 22 *l/s*, the *d*_*sm*_ was recorded as 0.037, 0.058, 0.074, 0.08, and 0.095 m, respectively. Conversely, as the flow rates receded (*Q*_*f*_) to 18, 14, 10, and 6 *l/s* during the falling limb, the corresponding *d*_*sm*_ decreased to 0.092, 0.089, 0.087, and 0.089 m. In the case of 15% blockage, a slight increase in the maximum scour depth was observed during the rising limb (*Q*_*r*_), resulting in values of 0.04, 0.073, 0.08, 0.09, and 0.1 m for flow rates of 6, 10, 14, 18, and *Q*_*M*_ = 22 *l/s*, respectively. This decrease persisted during the falling limb (*Q*_*f*_), with scour depths reaching 0.102, 0.104, 0.104, and 0.108 m. For the 30% blockage case, an increase in flow rate was correlated with an increase in *d*_*sm*_. During the rising limb, maximum scour depths were 0.049, 0.072, 0.09, 0.095, and 0.11 m. These values continued to rise during the falling limb, with recorded depths of 0.117, 0.12, 0.123, and 0.124 m. In summary, the 15% blockage condition resulted in greater scour depths compared to the non-blocked case, while the 30% blockage led to even higher scour depths than both previous cases. These findings underscore the intricate relationship between flow rate and blockage in shaping scour patterns.

In the case of a circular culvert under the same hydrograph conditions, an increase in flow rate resulted in a gradual escalation of scour depth. Specifically, for flow rates of 6, 10, 14, 18, and *Q*_*M*_ = 22 *l/s*, the recorded scour depths were 0.066, 0.089, 0.098, 0.10, and 0.105 m, respectively. During the falling limb (*Q*_*f*_) of the hydrograph, the *d*_*sm*_ values exhibited some fluctuations, yet remained consistently. In the presence of a 15% blockage, the rate of *d*_*sm*_ increased in tandem with the flow rate. Specifically, for flow rates of 6, 10, 14, 18, 22, and *Q*_*f*_ = 18 *l/s*, the corresponding *d*_*sm*_ values were 0.062, 0.088, 0.093, 0.107, 0.114, and 0.119 m. During the falling limb (*Q*_*f*_), the *d*_*sm*_ values decreased by 0.115 m. Similarly, the *d*_*sm*_ a continuous increase for the 30% blockage case. During the rising limb, the scour depths were recorded at 0.087, 0.10, 0.105, and 0.108 m, while at *Q*_*M*_ = 22 *l/s*, it reached 0.12 m. The corresponding *d*_*sm*_ values were approximately 0.124, 0.128, 0.127, and 0.132 m during the falling limb. In summary, the overall results highlight that an increase in the inlet blockage led to higher *d*_*sm*_. Also, indicate that for the first hydrograph, the *d*_*sm*_ in the circular culvert exceeded that in the box culvert, demonstrating the distinctive behaviour of circular culverts in response to varying flow rates and blockage levels [20].

**Fig 8:**
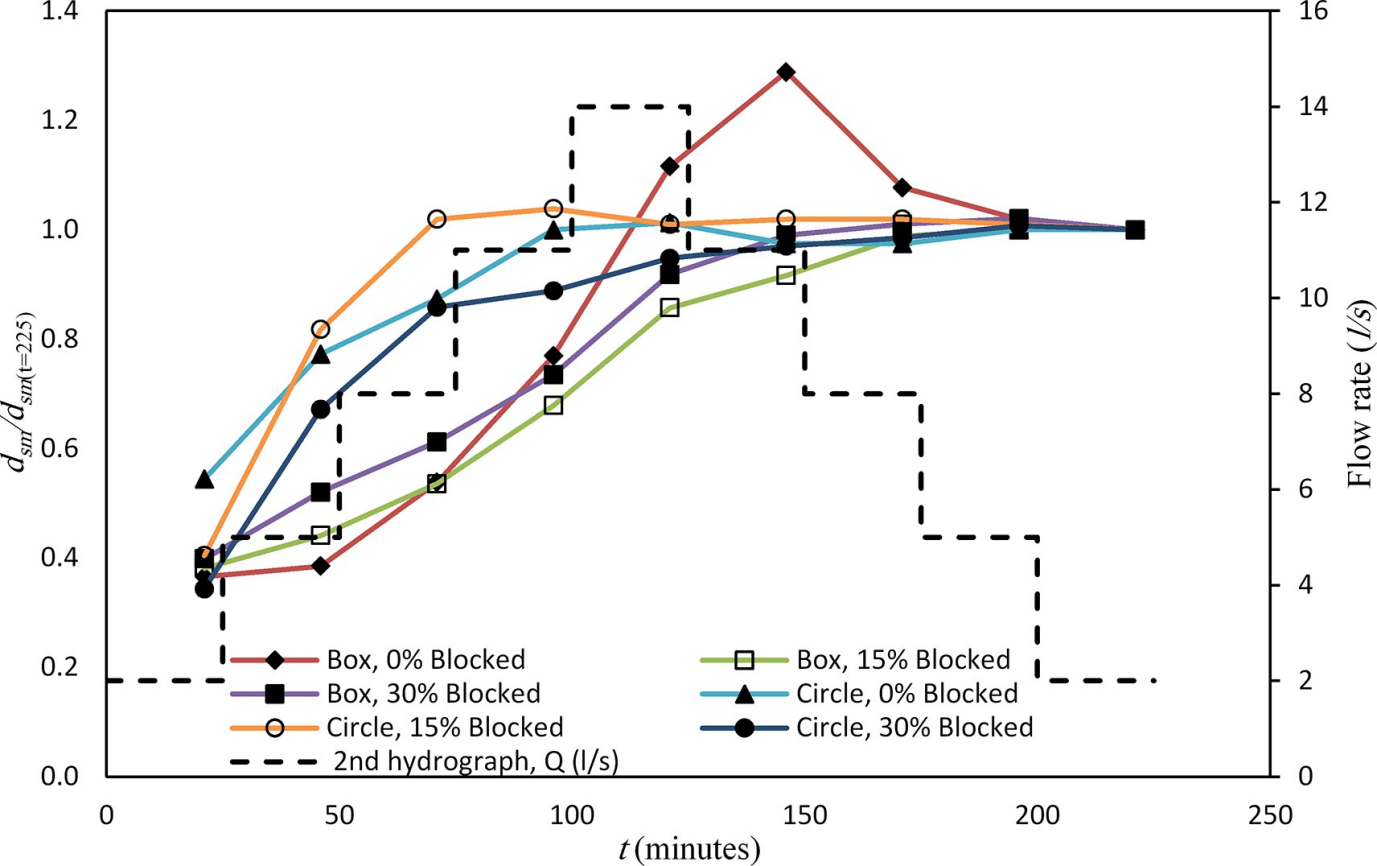
Comparison of *d*_*sm*_ between box and circular culvert of second hydrograph (*Q*_*M*_ = 14 *l/s*).

Fig 8 provides a visual representation of the *d*_*sm*_ for both types of culverts, considering various rates of blockages and the *t* = 225 min, with an initial flow rate ranging from *Q*_*r*_ = 2 *l/s* to *Q*_*M*_ = 14 *l/s* and then falling the limb to *Q*_*f*_ = 2 *l/s* for the second hydrograph. In the case of the box culvert, for flow rates of *Q*_*r*_ = 2, 5, 8, 11, *l/s* and *Q*_*M*_ = 14*l/s*, the scour depths gradually increased, measuring 0.019, 0.020, 0.028, 0.040, 0.058, and 0.067 m, respectively. During the falling limb (*Q*_*f*_), as the flow rates decreased, the *d*_*sm*_ decreased. For the 15% blockage case with the same stepwise hydrograph, the *d*_*sm*_ values increased accordingly. It reached a maximum *d*_*sm*_ = 0.072 m at *Q*_*M*_ = 14 *l/s*. Increasing the rate of inlet blockage to 30% led to an overall increase in *d*_*sm*_ values, and proportionally changed with the flow rate.

In the case of the circular culvert utilizing the same stepwise hydrograph, an increase in the rate of inlet blockage consistently led to higher scour depths. Specifically, in the non-blocked case, the *d*_*sm*_ values for the first four flow rates were approximately 0.043, 0.061, 0.069, and 0.079 m, with *d*_*sm*_ = 0.08 m corresponding to *Q*_*M*_ = 14 *l/s*. However, this increase did not persist during the falling limb, as the values remained relatively stable around 0.077 m. In the presence of a 15% inlet blockage, the *d*_*sm*_ values exhibited a noticeable increase, starting from the initial fourth *Q*_*r*_ with values of about 0.042, 0.085, 0.106, and 0.108 m, respectively. For the remaining discharge rates from *Q*_*M*_ *=* 14 *l/s* to *Q*_*f*_ *= 2 l/s*, the *d*_*sm*_ values fluctuated slightly, averaging around 0.105, 0.106, 0.106, 0.105, and 0.104 m. In the case of a 30% inlet blockage, the *d*_*sm*_ values consistently increased for all stepwise changes in the hydrograph except for the last stepwise change.

In summary, these findings reveal that the *d*_*sm*_ was generally more significant in the circular culvert compared to the box culvert for both hydrographs, and the rate of blockage had a proportional effect on increasing the *d*_*sm*_ in the circular culvert more than the box culvert [7].

## Conclusion

Scour development during flooding is a common problem that can cause hydraulic structure failure. In this research, scouring downstream of the box and circular culvert outlets was recorded under steady and unsteady water flow conditions under clear water with different blockage rates: 0%, 15%, and 30%. The most important results obtained from this research can be described as follows:

1. For high flow condition, the box culvert shows a direct correlation between blockage rate and maximum scour depths. A 15% inlet blockage led to a 13.7% increase in scour depths, while a 30% inlet blockage resulted in a roughly 23.4% increase compared to the non-blocked condition. Furthermore, the result of circular culvert with a 30% blockage when compared to 15% blockage and no blockage results in a significant increase of 9.8% and 15.9% in the maximum scour depth, respectively. Overall, the scour profile exhibited more growth in the circular culvert as compared to the box culvert across all cases.
2. For low flow in unsteady condition, the findings for box culvert indicated that the presence of a 30% inlet blockage has a considerably greater influence, the scour increased by the rate of 16% and 42% compared to the 15% and no blockage, respectively. Whenever, for circular culvert, 30% blockage has more significant effect on the formation of scouring. Additionally, the rate of change of 30% inlet blockage increases in scouring and observed to be 21% and 41% when compared to the 15% inlet blocked and nonblocked cases, respectively.
3. The maximum rate of flow 14 l/s and 22 l/s used in both steady cases. It was observed an increase in flow rate from 14 l/s to 22 l/s the maximum scour hole size and the maximum scour depth along the downstream section of the culvert increased. Conversely, when increased the degree of blockage, it was not observing a strictly proportional increase in scour depth across all cases. Instead, it was observed variations, with some cases showing increased scour depths.
4. The scouring depth and maximum scour depth increased with a rise in hydrograph stepwise and blockage rate. When comparing the same rate of blockage and non-blockage, it was found that the circular culvert indicated more scouring depth than the box culvert.

Once again, these observations affirm that an increase in the rate of inlet blockage does not necessarily lead to a higher degree of scouring. It is also essential to acknowledge the limitations of experimental studies, particularly the challenges posed by the small-dimension flume and the difficulty in accurately distinguishing between contraction scour and local scour, which may affect the data. A wider flume would likely result in more accurate data and is recommended for future research.

## Supporting information

S1 File The data set used in this research, in steady and unsteady flow conditions. (PDF)

## Abbreviations

*d_50_*: average sediments grain size
*σ_g_*: geometric standard deviation
$u_{{*}_{c}}$: critical shear velocity
*V_c_*: critical flow velocity
*V*: average of flow velocity
*y_w_*: water depth
*R_e_*: Reynolds numbers
*Q*: flow rate
*Q_r_*: rising flow rate in hydrographs
*Q_M_*: maximum flow rate
*Q_f_*: falling flow rate
*W*: width of the upstream channel
*S_0_*: bed slope
*D*: diameter
*F*: feeding rate of woody debris into the flow
*T*: time
*ρ*: fluid density
*ρ_w_*: wooden debris density
*μ*: dynamic viscosity of water
*g*: acceleration due to gravity
*H_e_*: the flow depth at the culvert inlet
*H_u_*: the upstream flow depth
*L_w_*: length of wooden debris
*h_c_*: culvert width
*L_c_*: culvert length
*X*: the distance between the accumulated debris and the channel bed at the inlet
*A_w_*: area of the culvert opening occupied by the wooden debris
*A_c_*: total area of the culvert opening
*B*: rate of blockage
*X**: non-dimensional blockage height
*F_re_*: Froude number at the culvert entrance
*F*_*ru*_: upstream flow Froude number
*d_s_*: actual scour depth
*d_sm_*: the maximum scour depth
