# Correction: Physical modeling of the effect of shape, blockage, and flow variability on scour in culvert outlets

**DOI:** 10.1371/journal.pone.0316589

**Published:** 2024-12-26

**Authors:** Kaywan Othman Ahmed, Mohammad Reza Kavianpour, Ata Amini, Younes Aminpour

There is an error in affiliation 2 for author Kaywan Othman Ahmed. The correct affiliation 2 is: Civil Engineering Department, Tishk International University, Sulaimani, Iraq.
