# Supplementary material for: Physical modeling of the effect of shape, blockage, and flow variability on scour in culvert outlets
Source: PLoS One. 2024 Jun 27;19(6):e0306252. doi: 10.1371/journal.pone.0306252 (PMC11210847; doi:10.1371/journal.pone.0306252)
Supplement: S1 File — (PDF) [file pone.0306252.s001.pdf]

The first hydrograph; Unsteady flow conditions; Box culvert; blockage 0%

Box, Unsteady, First Hydrograph, 360 min 0% Blockage

**Distance (cm)**	**Distance (mm)**	6 lit/s or 21.6 m3/h, **40 min**	10 lit/s or 36 m3/h, **80 min**	14 lit/s 50.4 m3/h, **120 min**	18 lit/s or 64.8 m3/h, **160 min**	22lit/s 79.2 m3/h, **200 min**	18 lit/s or 64.8 m3/h, **240 min**	14 lit/s 50.4 m3/h, **280 min**	10 lit/s or 36 m3/h, **320 min**	6 lit/s or 21.6 m3/h, **360 min**
0	0	-7	-12	-21	-29	-30	-33	-37	-37	-39
2	20	-22	-34	-39	-43	-36	-43	-41	-40	-46
4	40	-24	-41	-55	-46	-40	-46	-51	-47	-52
6	60	-30	-43	-57	-65	-70	-64	-65	-52	-49
8	80	-34	-53	-64	-69	-78	-63	-63	-56	-57
10	100	-34	-56	-60	-67	-80	-65	-65	-53	-62
12	120	-37	-54	-65	-72	-73	-64	-65	-65	-66
14	140	-33	-58	-61	-76	-76	-73	-68	-74	-69
16	160	-31	-58	-73	-74	-81	-72	-77	-73	-72
18	180	-27	-50	-74	-71	-85	-80	-84	-75	-73
20	200	-26	-51	-74	-75	-86	-83	-80	-80	-77
22	220	-23	-49	-72	-80	-85	-83	-81	-78	-81
24	240	-17	-50	-70	-78	-90	-85	-80	-78	-82
26	260	-16	-50	-72	-76	-95	-82	-83	-82	-80
28	280	-16	-48	-70	-77	-91	-88	-89	-83	-81
30	300	-13	-43	-65	-70	-83	-85	-84	-83	-89
32	320	-11	-43	-63	-66	-87	-89	-88	-87	-79
34	340	-14	-38	-58	-68	-86	-92	-80	-86	-73
36	360	-13	-35	-61	-72	-93	-87	-84	-85	-76
38	380	-11	-38	-55	-68	-88	-86	-79	-83	-79
40	400	-7	-35	-56	-66	-87	-87	-87	-85	-83
42	420	-7	-38	-52	-63	-88	-79	-78	-84	-86
44	440	-3	-30	-42	-57	-76	-73	-80	-77	-74
46	460	-4	-29	-46	-55	-83	-70	-72	-76	-66
48	480	-4	-26	-53	-65	-70	-62	-66	-75	-62
50	500	-6	-18	-41	-65	-68	-60	-64	-80	-60
52	520	-5	-17	-40	-67	-70	-58	-57	-78	-51
54	540	-3	-17	-37	-57	-65	-56	-60	-80	-51
56	560	-2	-16	-34	-44	-60	-44	-51	-59	-55
58	580	0	-25	-23	-42	-63	-40	-47	-53	-48
60	600	0	-8	-17	-38	-51	-40	-43	-41	-44
62	620		0	-15	-33	-43	-37	-37	-34	-38
64	640		5	-18	-27	-42	-34	-38	-27	-31
66	660		3	-7	-22	-40	-33	-31	-25	-23
68	680		12	-5	-17	-35	-30	-27	-20	-23
70	700		12	5	-12	-28	-17	-23	-15	-18
72	720		16	5	-7	-23	-13	-24	-15	-15
74	740		19	13	2	-13	-6	-14	-6	-7
76	760		13	16	0	-13	-9	-6	-4	-5
78	780		15	23	3	-7	0	-10	-2	-1
80	800		17	22	6	-3	7	-1	2	-2
82	820		13	30	9	-3	9	0	5	4
84	840		7	35	7	5	7	0	5	5
86	860		6	37	13	4	12	8	7	6
88	880		5	28	13	10	20	10	13	9
90	900		3	25	19	16	23	14	16	14
92	920		3	19	23	16	25	23	23	19
94	940		-2	20	25	22	19	22	24	20
96	960		-12	19	29	27	20	26	25	25
98	980		0	15	30	29	23	22	28	27
100	1000			15	39	27	30	21	31	28
102	1020			0	35	25	30	29	32	30
104	1040				34	26	29	27	30	34
106	1060				35	24	26	29	29	30
108	1080				30	23	28	27	28	27
110	1100				25	23	28	30	29	28
112	1120				20	23	25	29	28	26
114	1140				17	29	27	29	29	33
116	1160				13	30	26	30	27	30
118	1180					34	24	30	23	27
120	1200					29	20	30	24	28
122	1220					29	20	27	23	22
124	1240					20	23	24	22	24
126	1260					20	17	25	21	24
128	1280					10	20	23	20	17
130	1300					12	14	21	17	19
132	1320					13	10	17	12	19
134	1340					0	5	15	0	14
136	1360						5	13		10
138	1380						0	11		14
140	1400							0		15
142	1420									12
144	1440									0
146	1460									
148	1480									
150	1500									

The first hydrograph; Unsteady flow conditions; Box culvert; blockage 15%

Box, Unsteady, First Hydrograph, 360 min, 15% Blockage										
**Distance (cm)**	**Distance (mm)**	**6 lit/s or 21.6 m3/h, 40 min**	10 lit/s or 36 m3/h, **80 min**	14 lit/s 50.4 m3/h, **120 min**	18 lit/s or 64.8 m3/h, **160 min**	22lit/s 79.2 m3/h, **200 min**	18 lit/s or 64.8 m3/h, **240 min**	14 lit/s 50.4 m3/h, **280 min**	10 lit/s or 36 m3/h, **320 min**	6 lit/s or 21.6 m3/h, **360 min**
0	0	-9	-12	-22	-33	-37	-36	-38	-38	-41
2	20	-19	-23	-37	-43	-42	-38	-45	-38	-48
4	40	-20	-34	-43	-42	-40	-55	-53	-47	-52
6	60	-30	-43	-61	-55	-60	-70	-60	-56	-58
8	80	-33	-42	-40	-56	-62	-69	-64	-56	-60
10	100	-40	-46	-50	-66	-72	-70	-64	-60	-62
12	120	-31	-48	-58	-76	-70	-81	-71	-64	-66
14	140	-30	-50	-53	-75	-82	-80	-68	-66	-68
16	160	-24	-55	-55	-83	-81	-82	-72	-66	-72
18	180	-22	-57	-62	-80	-93	-82	-75	-72	-76
20	200	-30	-62	-63	-81	-94	-83	-83	-77	-77
22	220	-16	-70	-56	-85	-94	-87	-81	-80	-80
24	240	-16	-73	-58	-78	-86	-80	-85	-81	-81
26	260	-14	-71	-56	-82	-87	-90	-81	-83	-84
28	280	-16	-69	-60	-75	-93	-96	-80	-82	-83
30	300	-12	-68	-57	-75	-86	-102	-87	-82	-85
32	320	-15	-52	-63	-89	-91	-95	-95	-81	-97
34	340	-16	-58	-68	-90	-90	-86	-110	-95	-108
36	360	-14	-52	-70	-83	-90	-88	-104	-104	-87
38	380	-12	-45	-75	-71	-100	-83	-98	-90	-80
40	400	-20	-51	-80	-63	-83	-78	-85	-81	-76
42	420	-13	-41	-77	-63	-82	-84	-83	-80	-76
44	440	-20	-36	-54	-61	-85	-75	-73	-78	-73
46	460	-15	-30	-44	-61	-82	-73	-72	-75	-66
48	480	-7	-23	-36	-57	-78	-73	-68	-71	-66
50	500	-8	-17	-36	-60	-79	-71	-67	-78	-70
52	520	-10	-16	-29	-43	-68	-68	-63	-65	-58
54	540	-7	-17	-27	-36	-67	-60	-56	-57	-56
56	560	-5	-13	-13	-37	-61	-56	-64	-53	-55
58	580	-3	-9	-19	-33	-56	-54	-48	-47	-50
60	600	-3	-6	-17	-31	-53	-54	-47	-45	-42
62	620	-2	0	-18	-24	-44	-48	-46	-50	-37
64	640	0	3	-10	-16	-38	-38	-38	-35	-32
66	660		0	-15	-12	-35	-32	-30	-30	-27
68	680		3	-18	-11	-26	-24	-36	-27	-25
70	700		4	-15	-3	-20	-20	-23	-20	-23
72	720		7	-9	-1	-20	-13	-15	-19	-22
74	740		13	-6	0	-15	-14	-24	-17	-7
76	760		14	-6	-2	-6	-6	-22	-7	-6
78	780		15	-4	-1	0	-3	-6	-5	-3
80	800		17	-7	5	2	1	-5	-3	1
82	820		16	-3	1	0	4	3	0	2
84	840		19	-2	9	0	5	4	3	7
86	860		13	0	9	12	13	10	10	14
88	880		24	10	14	21	16	11	11	24
90	900		26	11	15	24	22	19	19	24
92	920		26	17	17	21	24	20	25	27
94	940		21	17	16	28	25	22	24	25
96	960		20	21	21	28	30	22	24	29
98	980		11	19	24	27	27	27	24	27
100	1000		13	20	29	28	24	29	27	30
102	1020		14	24	25	21	29	34	24	34
104	1040		17	23	25	26	28	35	30	32
106	1060		22	25	22	25	34	33	28	33
108	1080		7	20	23	24	33	33	32	35
110	1100		0	27	25	27	33	32	30	34
112	1120			34	26	25	35	32	34	37
114	1140			36	25	29	38	31	32	32
116	1160			30	31	30	36	30	34	34
118	1180			35	28	33	33	33	32	34
120	1200			37	29	34	34	36	30	35
122	1220			37	28	34	37	36	35	30
124	1240			36	34	33	33	32	35	27
126	1260			35	33	33	33	28	37	26
128	1280			37	32	30	31	25	39	27
130	1300			37	35	30	31	22	35	20
132	1320			34	30	29	24	23	34	24
134	1340			31	32	27	24	23	32	22
136	1360			27	35	24	29	17	33	25
138	1380			23	29	27	27	15	30	25
140	1400			14	25	29	25	12	28	22
142	1420			0		22	21	0	30	18
144	1440					21	17		24	17
146	1460					17	15		21	12
148	1480					14	12		18	0
150	1500					0	0		14	0

The first hydrograph; Unsteady flow conditions; Box culvert; blockage 30%

Box, Unsteady, First Hydrograph, 360 min, 30% Blockage										
**Distance (cm)**	**Distance (mm)**	6 lit/s or 21.6 m3/h, **40 min**	10 lit/s or 36 m3/h, **80 min**	14 lit/s 50.4 m3/h, **120 min**	18 lit/s or 64.8 m3/h, **160 min**	22lit/s 79.2 m3/h, **200 min**	18 lit/s or 64.8 m3/h, **240 min**	14 lit/s 50.4 m3/h, **280 min**	10 lit/s or 36 m3/h, **320 min**	6 lit/s or 21.6 m3/h, **360 min**
0	0	-12	-16	-21	-32	-35	-36	-38	-41	-41
2	20	-26	-30	-33	-42	-40	-40	-43	-42	-43
4	40	-40	-45	-48	-50	-50	-43	-43	-48	-47
6	60	-49	-55	-50	-56	-51	-52	-55	-53	-50
8	80	-29	-60	-53	-58	-60	-52	-56	-57	-55
10	100	-26	-65	-63	-61	-63	-75	-60	-60	-60
12	120	-25	-72	-70	-65	-80	-83	-63	-70	-70
14	140	-19	-63	-72	-73	-73	-90	-62	-71	-79
16	160	-24	-50	-80	-78	-83	-98	-73	-87	-87
18	180	-20	-43	-85	-80	-74	-105	-79	-96	-110
20	200	-20	-36	-90	-85	-77	-110	-90	-109	-118
22	220	-12	-34	-82	-87	-87	-108	-98	-118	-124
24	240	-13	-38	-78	-95	-93	-117	-102	-123	-119
26	260	-14	-34	-62	-88	-110	-102	-110	-118	-117
28	280	-13	-37	-50	-83	-95	-91	-112	-115	-116
30	300	-6	-30	-43	-72	-87	-80	-119	-112	-115
32	320	-7	-34	-42	-69	-76	-71	-120	-108	-117
34	340	-7	-26	-37	-65	-74	-63	-115	-100	-110
36	360	-5	-35	-30	-59	-68	-62	-112	-98	-108
38	380	-3	-23	-25	-50	-58	-60	-74	-56	-107
40	400	-2	-19	-24	-49	-65	-57	-70	-53	-102
42	420	-3	-19	-12	-42	-60	-56	-63	-47	-93
44	440	-2	-20	-10	-40	-53	-46	-50	-48	-82
46	460	-2	-20	-4	-48	-52	-45	-46	-40	-75
48	480	-5	-22	-2	-41	-45	-33	-43	-36	-62
50	500	0	-20	2	-35	-44	-37	-46	-31	-34
52	520		-15	11	-28	-33	-35	-36	-30	-33
54	540		-7	16	-25	-29	-27	-33	-24	-26
56	560		-5	17	-15	-31	-23	-27	-21	-24
58	580		-5	18	0	-17	-22	-20	-14	-20
60	600		-4	21	2	-15	-21	-13	-6	-11
62	620		0	23	4	-14	-6	-8	-5	-4
64	640			22	7	-10	-5	-7	-3	-1
66	660			17	11	-6	4	-3	5	-1
68	680			12	15	1	6	2	8	5
70	700			10	18	7	14	1	10	12
72	720			26	27	17	19	7	17	18
74	740			6	20	22	24	17	20	24
76	760			0	15	17	22	19	23	22
78	780				12	20	24	19	25	30
80	800				7	29	26	26	30	30
82	820				0	27	29	36	35	32
84	840					30	32	35	38	35
86	860					30	34	34	41	39
88	880					32	37	39	39	43
90	900					45	40	43	37	45
92	920					44	44	41	39	46
94	940					42	42	42	41	42
96	960					39	42	43	43	40
98	980					40	40	37	41	40
100	1000					35	38	35	38	39
102	1020					27	30	36	36	38
104	1040					19	31	30	32	36
106	1060					15	24	27	27	31
108	1080					15	19	23	25	28
110	1100					10	17	20	24	24
112	1120					6	12	18	20	21
114	1140					5	8	15	17	18
116	1160					0	6	13	14	12
118	1180						2	10	11	11
120	1200						0	8	9	9
122	1220							7	9	8
124	1240							5	8	5
126	1260							3	6	2
128	1280							1	2	0
130	1300							0	0	

The first hydrograph; Unsteady flow conditions; Circle culvert; blockage 0%

Circle, Unsteady, First Hydrograph, 360 min 0% Blockage

**Distance (cm)**	**Distance (mm)**	6 lit/s or 21.6 m3/h, **40 min**	10 lit/s or 36 m3/h, **80 min**	14 lit/s 50.4 m3/h, **120 min**	18 lit/s or 64.8 m3/h, **160 min**	22lit/s 79.2 m3/h, **200 min**	18 lit/s or 64.8 m3/h, **240 min**	14 lit/s 50.4 m3/h, **280 min**	10 lit/s or 36 m3/h, **320 min**	6 lit/s or 21.6 m3/h, **360 min**
0	0	-21	-29	-35	-39	-42	-47	-48	-50	-50
2	20	-43	-52	-56	-46	-54	-52	-50	-54	-54
4	40	-55	-64	-70	-60	-55	-55	-54	-57	-50
6	60	-53	-67	-62	-65	-62	-60	-61	-60	-57
8	80	-64	-69	-66	-69	-66	-69	-63	-62	-60
10	100	-56	-75	-74	-71	-69	-72	-66	-68	-62
12	120	-62	-82	-66	-74	-75	-76	-70	-71	-61
14	140	-66	-85	-75	-79	-80	-77	-70	-66	-66
16	160	-57	-80	-70	-77	-80	-73	-75	-67	-68
18	180	-58	-87	-82	-75	-82	-76	-74	-78	-72
20	200	-50	-73	-76	-72	-86	-79	-73	-80	-74
22	220	-61	-82	-75	-74	-88	-80	-80	-84	-77
24	240	-55	-77	-74	-77	-90	-86	-80	-86	-81
26	260	-53	-78	-77	-78	-90	-90	-88	-92	-87
28	280	-47	-85	-78	-76	-88	-96	-86	-94	-81
30	300	-59	-88	-81	-83	-91	-95	-97	-98	-86
32	320	-60	-89	-84	-81	-93	-100	-102	-96	-91
34	340	-55	-82	-89	-91	-97	-102	-106	-100	-93
36	360	-57	-79	-85	-86	-101	-104	-111	-105	-102
38	380	-62	-77	-90	-91	-100	-104	-106	-104	-106
40	400	-61	-83	-83	-87	-104	-107	-105	-102	-108
42	420	-60	-77	-95	-96	-100	-105	-105	-107	-107
44	440	-59	-79	-97	-98	-101	-104	-105	-105	-109
46	460	-50	-82	-98	-100	-105	-108	-104	-109	-111
48	480	-55	-85	-96	-100	-100	-110	-109	-110	-103
50	500	-53	-82	-95	-94	-102	-107	-106	-106	-100
52	520	-57	-83	-93	-98	-102	-103	-106	-108	-96
54	540	-57	-81	-88	-89	-104	-106	-102	-107	-95
56	560	-55	-71	-89	-96	-96	-105	-106	-105	-96
58	580	-56	-75	-84	-96	-95	-101	-104	-102	-92
60	600	-53	-65	-81	-94	-100	-100	-102	-101	-95
62	620	-45	-71	-76	-91	-95	-100	-101	-100	-91
64	640	-46	-63	-83	-88	-90	-99	-96	-106	-91
66	660	-46	-64	-77	-87	-90	-96	-95	-93	-90
68	680	-50	-62	-73	-83	-92	-94	-96	-89	-88
70	700	-40	-55	-75	-81	-94	-91	-94	-89	-85
72	720	-28	-51	-75	-80	-86	-93	-93	-70	-83
74	740	-22	-50	-71	-79	-88	-88	-88	-72	-81
76	760	-16	-46	-67	-74	-84	-85	-84	-65	-78
78	780	-17	-41	-65	-72	-79	-82	-79	-63	-71
80	800	-15	-40	-62	-71	-77	-77	-77	-65	-71
82	820	-7	-36	-58	-70	-81	-75	-74	-66	-71
84	840	1	-36	-57	-70	-80	-72	-67	-60	-71
86	860	4	-22	-55	-68	-72	-67	-70	-56	-68
88	880	6	-25	-52	-65	-65	-68	-65	-52	-66
90	900	8	-13	-50	-61	-57	-63	-60	-50	-63
92	920	18	-10	-51	-63	-60	-56	-59	-45	-65
94	940	11	-12	-45	-60	-61	-57	-53	-41	-58
96	960	18	-9	-39	-55	-54	-50	-49	-35	-56
98	980	28	-5	-33	-49	-57	-47	-45	-35	-53
100	1000	27	-5	-37	-43	-55	-43	-42	-30	-51
102	1020	25	-3	-37	-43	-40	-42	-40	-26	-50
104	1040	21	0	-30	-42	-36	-34	-30	-24	-48
106	1060	18	1	-27	-38	-29	-32	-26	-20	-43
108	1080	23	1	-27	-30	-25	-27	-26	-17	-42
110	1100	18	5	-10	-33	-20	-22	-20	-13	-38
112	1120	15	9	-8	-30	-13	-21	-16	-9	-32
114	1140	11	10	-3	-22	-7	-20	-11	-3	-27
116	1160	12	3	3	-17	-5	-19	-6	-1	-15
118	1180	9	9	5	-13	-4	-10	-1	1	-12
120	1200	5	10	7	-8	3	-4	0	15	-10
122	1220	0	17	10	2	10	-2	4	11	-9
124	1240		23	8	5	15	4	10	16	-5
126	1260		30	7	6	25	5	15	21	-1
128	1280		28	14	9	21	4	18	25	0
130	1300		32	17	6	21	15	19	27	7
132	1320		35	20	10	30	17	22	32	9
134	1340		30	25	19	30	21	24	36	15
136	1360		34	33	18	36	24	22	34	18
138	1380		36	32	21	38	27	34	33	22
140	1400		35	40	23	43	29	36	38	36
142	1420		34	39	25	43	35	34	50	39
144	1440		31	40	26	50	34	43		36
146	1460		30	44	30		44	41		36
148	1480		28	39	30		47			34
150	1500		23	32	33		50			32

The first hydrograph; Unsteady flow conditions; Circle culvert; blockage 15%

Circle, Unsteady, First Hydrograph, 360 min, 15% Blockage

**Distance (cm)**	**Distance (mm)**	6 lit/s or 21.6 m3/h, **40 min**	10 lit/s or 36 m3/h, **80 min**	14 lit/s 50.4 m3/h, **120 min**	18 lit/s or 64.8 m3/h, **160 min**	22lit/s 79.2 m3/h, **200 min**	18 lit/s or 64.8 m3/h, **240 min**	14 lit/s 50.4 m3/h, **280 min**	10 lit/s or 36 m3/h, **320 min**	6 lit/s or 21.6 m3/h, **360 min**
0	0	-7	-32	-39	-42	-49	-52	-53	-59	-62
2	20	-29	-62	-66	-65	-65	-69	-64	-71	-70
4	40	-40	-60	-61	-67	-67	-67	-69	-73	-60
6	60	-46	-65	-64	-66	-68	-66	-67	-69	-61
8	80	-48	-73	-62	-71	-80	-70	-70	-69	-62
10	100	-53	-72	-65	-72	-81	-75	-72	-72	-66
12	120	-55	-78	-63	-71	-85	-80	-80	-75	-66
14	140	-60	-75	-74	-73	-87	-77	-81	-77	-67
16	160	-62	-78	-78	-74	-88	-76	-84	-78	-70
18	180	-62	-73	-78	-75	-90	-80	-82	-84	-74
20	200	-60	-77	-74	-81	-91	-83	-85	-83	-76
22	220	-58	-72	-76	-82	-93	-87	-87	-85	-80
24	240	-57	-80	-77	-84	-96	-86	-90	-90	-81
26	260	-53	-80	-78	-85	-102	-88	-95	-91	-85
28	280	-50	-78	-81	-93	-106	-93	-95	-93	-87
30	300	-53	-81	-84	-96	-102	-100	-100	-95	-90
32	320	-50	-84	-86	-98	-110	-104	-102	-96	-93
34	340	-53	-87	-90	-97	-107	-106	-103	-103	-95
36	360	-57	-84	-92	-95	-102	-103	-105	-107	-97
38	380	-53	-85	-93	-107	-102	-110	-105	-110	-102
40	400	-50	-83	-90	-102	-108	-108	-110	-110	-106
42	420	-49	-83	-89	-104	-109	-105	-110	-108	-115
44	440	-40	-88	-90	-100	-108	-110	-110	-109	-114
46	460	-37	-87	-87	-99	-116	-114	-118	-114	-109
48	480	-36	-87	-90	-98	-114	-118	-113	-112	-110
50	500	-39	-85	-87	-101	-112	-119	-110	-113	-108
52	520	-35	-83	-85	-104	-106	-113	-107	-110	-106
54	540	-32	-80	-86	-102	-106	-113	-107	-109	-102
56	560	-29	-82	-81	-100	-106	-110	-106	-108	-106
58	580	-27	-76	-80	-102	-95	-110	-102	-106	-106
60	600	-23	-73	-85	-102	-86	-107	-105	-109	-109
62	620	-23	-72	-83	-101	-86	-104	-102	-108	-102
64	640	-22	-73	-77	-97	-88	-105	-99	-105	-105
66	660	-27	-72	-73	-95	-89	-100	-96	-102	-98
68	680	-20	-72	-73	-94	-87	-96	-95	-95	-97
70	700	-13	-72	-77	-92	-82	-96	-90	-96	-94
72	720	-16	-73	-77	-93	-76	-90	-93	-97	-91
74	740	-13	-65	-73	-88	-79	-93	-90	-92	-89
76	760	-13	-64	-72	-86	-75	-88	-79	-86	-90
78	780	-8	-61	-67	-82	-72	-80	-78	-84	-85
80	800	-5	-58	-62	-79	-68	-78	-76	-78	-84
82	820	-4	-54	-63	-75	-61	-74	-68	-77	-80
84	840	-10	-55	-58	-76	-65	-70	-68	-71	-77
86	860	-7	-52	-62	-73	-56	-63	-61	-66	-73
88	880	-7	-48	-58	-67	-50	-61	-59	-63	-68
90	900	-3	-46	-53	-62	-44	-60	-57	-60	-64
92	920	-2	-46	-47	-58	-41	-47	-55	-55	-61
94	940	3	-40	-46	-53	-42	-42	-46	-56	-58
96	960	3	-34	-44	-53	-38	-41	-38	-47	-55
98	980	5	-30	-38	-49	-27	-33	-35.9	-43	-52
100	1000	0	-27	-33	-44	-29	-27	-29	-38	-45
102	1020		-16	-33	-40	-26	-24	-28	-37	-41
104	1040		-22	-29	-34	-22	-12	-23	-31	-38
106	1060		-14	-23	-31	-16	-26	-17	-27	-34
108	1080		-14	-16	-27	-4	-11	-13	-23	-31
110	1100		-12	-15	-22	-2	-5	-8	-16	-26
112	1120		-6	-7	-17	6	-4	-6	-17	-20
114	1140		-4	-6	-7	4	1	0	-7	-19
116	1160		-2	-1	-3	12	5	8	-3	-16
118	1180		7	6	-1	17	14	12	5	-8
120	1200		7	8.1	2	24	24	16	9	-4
122	1220		12	11	6	29	24	20	15	4
124	1240		14	12	10	36	26	26	22	10
126	1260		21	16	15	40	34	30	26	14
128	1280		20	18	19	45	40	34	34	17
130	1300		24	22	24	48	44	40	36	21
132	1320		25	21	28	55	45	47	40	29
134	1340		27	28	33	57	49	48	44	31
136	1360		34	30	38	60	50	53	50	36
138	1380		34	34	36			56	50	43
140	1400		34	37	38			60	62	44
142	1420		36	38	41					50
144	1440		32	39	43					55
146	1460		30	42	46					64
148	1480			46						70

The first hydrograph; Unsteady flow conditions; Circle culvert; blockage 30%

Circle, Unsteady, First Hydrograph, 360 min, 30% Blockage										
**Distance (cm)**	**Distance (mm)**	6 lit/s or 21.6 m3/h, **40 min**	10 lit/s or 36 m3/h, **80 min**	14 lit/s 50.4 m3/h, **120 min**	18 lit/s or 64.8 m3/h, **160 min**	22lit/s 79.2 m3/h, **200 min**	18 lit/s or 64.8 m3/h, **240 min**	14 lit/s 50.4 m3/h, **280 min**	10 lit/s or 36 m3/h, **320 min**	6 lit/s or 21.6 m3/h, **360 min**
0	0	-25	-38	-42	-49	-56	-60	-61	-62	-62
2	20	-60	-76	-71	-76	-74	-68	-68	-70	-68
4	40	-68	-78	-70	-76	-74	-73	-71	-69	-73
6	60	-67	-75	-66	-76	-77	-72	-71	-64	-67
8	80	-74	-73	-66	-74	-76	-70	-70	-72	-68
10	100	-79	-75	-65	-74	-80	-76	-72	-76	-69
12	120	-82	-75	-72	-73	-84	-73	-74	-74	-71
14	140	-87	-80	-73	-77	-84	-75	-76	-72	-72
16	160	-83	-83	-76	-78	-85	-77	-76	-75	-80
18	180	-87	-81	-77	-82	-85	-76	-75	-81	-84
20	200	-86	-77	-80	-85	-89	-84	-83	-82	-90
22	220	-80	-78	-80	-83	-88	-85	-86	-88	-98
24	240	-86	-81	-80	-88	-92	-86	-85	-93	-100
26	260	-83	-82	-82	-90	-93	-90	-94	-96	-104
28	280	-82	-80	-83	-91	-92	-91	-97	-99	-110
30	300	-77	-81	-86	-93	-100	-97	-101	-102	-118
32	320	-80	-83	-90	-95	-107	-98	-110	-105	-120
34	340	-75	-84	-91	-108	-110	-100	-109	-112	-126
36	360	-76	-84	-92	-95	-120	-108	-117	-106	-130
38	380	-74	-90	-90	-91	-108	-114	-126	-115	-132
40	400	-76	-100	-90	-90	-96	-120	-128	-127	-115
42	420	-75	-87	-98	-90	-86	-123	-120	-120	-110
44	440	-73	-87	-105	-90	-88	-124	-107	-108	-105
46	460	-77	-85	-100	-91	-92	-118	-100	-98	-107
48	480	-72	-81	-86	-90	-87	-106	-99	-100	-104
50	500	-72	-78	-85	-87	-83	-98	-99	-96	-98
52	520	-72	-75	-81	-87	-85	-97	-99	-92	-92
54	540	-72	-76	-80	-86	-81	-96	-101	-91	-91
56	560	-67	-74	-81	-84	-78	-91	-95	-90	-91
58	580	-66	-73	-80	-88	-80	-90	-86	-91	-83
60	600	-69	-72	-77	-89	-77	-82	-83	-85	-80
62	620	-63	-68	-76	-84	-75	-84	-77	-83	-80
64	640	-64	-68	-76	-78	-76	-79	-73	-76	-75
66	660	-63	-57	-73	-76	-72	-72	-69	-75	-74
68	680	-63	-60	-68	-76	-69	-70	-69	-74	-72
70	700	-63	-57	-63	-72	-68	-68	-66	-71	-70
72	720	-64	-57	-62	-74	-64	-66	-56	-62	-57
74	740	-61	-57	-64	-70	-63	-64	-59	-64	-56
76	760	-57	-49	-61	-66	-60	-62	-57	-59	-54
78	780	-53	-48	-57	-67	-57	-57	-52	-57	-52
80	800	-49	-43	-52	-62	-55	-55	-52	-52	-51
82	820	-46	-41	-50	-57	-55	-53	-47	-44	-48
84	840	-42	-38	-48	-56	-53	-50	-44	-43	-45
86	860	-39	-39	-46	-55	-46	-43	-37	-37	-42
88	880	-36	-34	-43	-51	-47	-39	-35	-35	-39
90	900	-33	-30	-38	-46	-42	-34	-35	-34	-36
92	920	-33	-27	-34	-47	-42	-33	-34	-30	-31
94	940	-27	-23	-36	-42	-40	-34	-27	-27	-27
96	960	-21	-21	-30	-37	-38	-29	-27	-24	-25
98	980	-15	-13	-27	-35	-37	-27	-25	-22	-26
100	1000	-6	-11	-24.9	-34	-33	-25	-27	-20	-20
102	1020	-6	-4	-28	-35	-30	-23	-22	-16	-17
104	1040	0	-2	-20	-35	-27	-13	-17	-13	-17
106	1060		-2	-12	-27	-26	-13	-13	-15	-14
108	1080		1	-17	-25	-23	-13	-13	-10	-11
110	1100		4	-10	-20	-20	-10	-8	-5	-9
112	1120		7	-5	-14	-22	-13	-7	-2	-7
114	1140		5	-1	-9	-18	-7	-4.9	4	-5
116	1160		10	6	-9	-14	-7	-1	2	2
118	1180		13	10	-4	-17	-5	0	4	2
120	1200		13	12	-2	-9	-7	6	8	3
122	1220		22	15	2	3	-2	5	10	5
124	1240		24	17	6		2	6	14	9
126	1260		27	25	14		5	13	18	11
128	1280		31	26	14		10		25	13
130	1300		33	28	18				30	15
132	1320		37	33	20					21
134	1340		39	40						26
136	1360		43							25
138	1380									34
140	1400									33
142	1420									40

The second hydrograph; Unsteady flow conditions; Box culvert; blockage 0%

Box, Unsteady, second Hydrograph, 225 min 0% Blockage

**Distance (cm)**	**Distance (mm)**	2 lit/s or 7.2 m3/h, **25 min**	5 lit/s or 18 m3/h, **50 min**	8 lit/s 28.8 m3/h, **75 min**	11 lit/s or 39.6 m3/h, **100 min**	14 lit/s 50.4 m3/h, **125 min**	11 lit/s or 39.6 m3/h, **150 min**	8 lit/s 28.8 m3/h, **175 min**	5 lit/s or 18 m3/h, **200 min**	2 lit/s or 7.2 m3/h, **225 min**
0	0	-5	-9	-12	-20	-23	-30	-32	-33	-36
2	20	-17	-13	-20	-29	-31	-43	-41	-37	-36
4	40	-18	-15	-17	-31	-51	-50	-45	-38	-45
6	60	-19	-17	-21	-32	-55	-52	-46	-43	-47
8	80	-13	-18	-28	-37	-54	-43	-44	-45	-50
10	100	-12	-19	-23	-38	-54	-47	-50	-48	-44
12	120	-11	-19	-24	-34	-57	-47	-41	-46	-50
14	140	-8	-20	-17	-36	-55	-53	-47	-46.5	-50
16	160	-4	-17	-9	-40	-53	-49	-52	-48	-48
18	180	-6	-15	-7	-33	-58	-54	-53	-48	-50
20	200	-4	-6	-8	-34	-53	-53	-55	-53	-47
22	220	-3	-9	-7	-30	-51	-49	-55	-50	-52
24	240	-1	-6	-10	-25	-53	-50	-56	-48	-50
26	260	-3	-4	-3	-24	-50	-49	-53	-46	-44
28	280	-3	-6	-6	-20	-53	-53	-48	-50	-50
30	300	0	-1	-5	-20	-50	-51	-46	-52	-44
32	320	1	-3	-7	-17	-46	-44	-50	-53	-43
34	340	3	-7	-9	-17	-45	-47	-45	-49	-40
36	360	2	-3	-10	-16	-44	-43	-43	-43	-37
38	380	-3	-8	-7	-15	-40	-41	-41	-38	-35
40	400	2	-2	-7	-14	-43	-38	-37	-37	-31
42	420	0	0	-11	-19	-41	-33	-34	-35	-25
44	440	2	-7	-13	-19	-30	-32	-33	-32	-20
46	460	4	0	-8	-16	-28	-37	-27	-30	-23
48	480	1	4	-9	-20	-23	-25	-27	-24	-20
50	500	5	-1	-5	-22	-20	-22	-25	-20	-15
52	520	4	5	-13	-18	-17	-19	-23	-25	-10
54	540	0	4	-6	-20	-16	-13	-18	-12	-7
56	560		2	-5	-13	-15	-13	-15	-8	-8
58	580		3	0	-7	-7	-5	-8	-4	-3
60	600		10	-1	-7	-1	0	-5	-3	-1
62	620		7	-3	-8	2	5	0	0	-1
64	640		9	-8	-7	2	4	3	2	3
66	660		7	-3	-10	5	13	5	6	0
68	680		4	4	-3	7	15	9	9	12
70	700			3	3	12	13	12	13	17
72	720			5	10	17	17	16	15	19
74	740			1	12	13	19	17	20	20
76	760			7	9	17	16	19	20	18
78	780			0	13	20	16	22	22	19
80	800				6	22	14	19	18	24
82	820				2	21	14	25	15	19
84	840				3	17	16	17	12	13
86	860				-1	14	19	21	19	17
88	880				3	16	17	20	19	17
90	900				0	13	13	22	20	18
92	920					10	6	19	15	17
94	940					5	9	15	11	0
96	960					2	8	13	7	2
98	980					0	4	7	8	-2
100	1000					0	0	7	4	2
102	1020							4	3	
104	1040							3	0	
106	1060							0	0	
108	1080							3		
110	1100							-3		
112	1120							1		

The second hydrograph; Unsteady flow conditions; Box culvert; blockage 15%

Box, Unsteady, second Hydrograph, 225 min 15% Blockage										
**Distance (cm)**	**Distance (mm)**	2 lit/s or 7.2 m3/h, **25 min**	5 lit/s or 18 m3/h, **50 min**	8 lit/s 28.8 m3/h, **75 min**	11 lit/s or 39.6 m3/h, **100 min**	14 lit/s 50.4 m3/h, **125 min**	11 lit/s or 39.6 m3/h, **150 min**	8 lit/s 28.8 m3/h, **175 min**	5 lit/s or 18 m3/h, **200 min**	2 lit/s or 7.2 m3/h, **225 min**
0	0	-9	-10	-14	-19	-21	-27	-29	-32	-33
2	20	-25	-16	-27	-30	-30	-37	-35	-38	-37
4	40	-32	-19	-32	-44	-47	-35	-41	-43	-35
6	60	-30	-23	-40	-46	-55	-51	-55	-46	-40
8	80	-28	-26	-45	-54	-57	-56	-48	-48	-46
10	100	-20	-37	-43	-53	-60	-61	-50	-52	-50
12	120	-14	-24	-41	-50	-63	-56	-54	-52	-55
14	140	-12	-16	-38	-48	-65	-60	-65	-50	-60
16	160	-11	-14	-36	-55	-67	-57	-70	-54	-75
18	180	-7	-13	-34	-54	-62	-62	-78	-61	-84
20	200	-7	-11	-31	-57	-71	-61	-81	-70	-73
22	220	-5	-9	-39	-48	-70	-60	-83	-77	-70
24	240	-2	-9	-24	-46	-70	-70	-78	-84	-62
26	260	-3	-8	-21	-45	-72	-77	-70	-78	-55
28	280	-2	-7	-28	-37	-70	-68	-58	-70	-40
30	300	-2	-5	-26	-36	-70	-60	-57	-62	-43
32	320	-2	-3	-19	-34	-66	-56	-55	-56	-42
34	340	-1	-2	-19	-30	-63	-56	-53	-54	-46
36	360	-2	-2	-23	-35	-61	-50	-50	-52	-44
38	380	0	-1	-16	-43	-55	-47	-52	-46	-42
40	400		-1	-20	-33	-48	-47	-42	-46	-42
42	420		-1	-14	-35	-38	-48	-44	-46	-35
44	440		0	-14	-32	-40	-44	-38	-38	-34
46	460			-16	-25	-36	-42	-34	-40	-32
48	480			-6	-24	-36	-41	-28	-33	-31
50	500			-8	-23	-33	-36	-24	-29	-30
52	520			-9	-15	-32	-30	-22	-26	-20
54	540			-3	-7	-28	-26	-18	-23	-20
56	560			-5	-5	-15	-22	-27	-17	-16
58	580			2	1	-16	-20	-15	-16	-12
60	600			0	4	-12	-16	-13	-7	-6
62	620				4	-7	-10	-6	-6	-3
64	640				9	0	-10	-1	-4	-1
66	660				9	6	-4	0	-3	-1
68	680				13	9	-4	2	3	4
70	700				5	13	-2	7	6	6
72	720				7	14	4	10	8	14
74	740				7	15	7	12	11	10
76	760				9	15	8	14	13	15
78	780				3	16	14	15	12	17
80	800				0	18	14	17	13	20
82	820					16	13	15	15	20
84	840					17	14	17	22	21
86	860					12	18	19	18	20
88	880					13	15	19	19	22
90	900					13	16	19	13	21
92	920					10	10	14	7	17
94	940					7	10	7	10	14
96	960					4	8	8	8	7
98	980					0	8	0	4	7
100	1000						5		0	5
102	1020						1			3
104	1040						0			0

The second hydrograph; Unsteady flow conditions; Box culvert; blockage 30%

Box, Unsteady, second Hydrograph, 225 min 30% Blockage

**Distance (cm)**	**Distance (mm)**	2 lit/s or 7.2 m3/h, **25 min**	5 lit/s or 18 m3/h, **50 min**	8 lit/s 28.8 m3/h, **75 min**	11 lit/s or 39.6 m3/h, **100 min**	14 lit/s 50.4 m3/h, **125 min**	11 lit/s or 39.6 m3/h, **150 min**	8 lit/s 28.8 m3/h, **175 min**	5 lit/s or 18 m3/h, **200 min**	2 lit/s or 7.2 m3/h, **225 min**
0	0	-12	-13	-14	-22	-31	-30	-33	-33	-34
2	20	-20	-20	-10	-34	-45	-33	-40	-40	-36
4	40	-28	-30	-16	-30	-58	-37	-36	-41	-37
6	60	-39	-39	-30	-34	-59	-40	-40	-43	-46
8	80	-28	-48	-38	-36	-60	-41	-42	-39	-42
10	100	-20	-50	-47	-48	-57	-43	-44	-45	-40
12	120	-10	-51	-53	-60	-62	-45	-45	-43	-39
14	140	-5	-41	-60	-65	-64	-45	-44	-39	-44
16	160	-6	-32	-50	-70	-65	-46	-46	-41	-45
18	180	0	-23	-38	-72	-68	-45	-43	-47	-46
20	200		-10	-29	-65	-80	-49	-50	-50	-50
22	220		-5	-11	-48	-85	-48	-58	-57	-59
24	240		-3	-17	-35	-90	-60	-60	-68	-67
26	260		-4	-6	-23	-78	-62	-65	-79	-78
28	280		-8	-4	-18	-70	-70	-77	-80	-84
30	300		-7	-5	-7	-56	-75	-85	-83	-90
32	320		-11	-5	-6	-54	-87	-99	-88	-98
34	340		-7	-3	-6	-50	-97	-90	-97	-84
36	360		-10	-2	-5	-50	-80	-77	-100	-73
38	380		-6	-2	-3	-50	-68	-70	-88	-67
40	400		-3	-5	-6	-46	-60	-55	-73	-52
42	420		-5	-5	-2	-48	-51	-50	-64	-41
44	440		-6	-5	-7	-49	-43	-43	-60	-40
46	460		-3	-6	-6	-50	-56	-36	-57	-37
48	480		0	-5	-5	-43	-35	-36	-37	-34
50	500			-3	-5	-41	-28	-33	-36	-33
52	520			-2	-4	-46	-30	-31	-33	-27
54	540			0	-5	-39	-33	-29	-35	-26
56	560				-6	-36	-30	-25	-29	-20
58	580				0	-37	-29	-25	-29	-22
60	600					-33	-26	-21	-24	-19
62	620					-30	-26	-18	-26	-16
64	640					-26	-19	-19	-19	-8
66	660					-21	-15	-15	-17	-7
68	680					-19	-29	-16	-16	-9
70	700					-25	-24	-17	-18	-10
72	720					-20	-16	-11	-15	-10
74	740					-16	-10	-8	-11	-7
76	760					-17	-8	-9	-24	-4
78	780					-19	-8	-10	-7	-3
80	800					-12	-8	-5	-6	-2
82	820					-19	-3	-5	-5	-2
84	840					-18	-1	-2	-1	-5
86	860					-15	-3	0	1	1
88	880					-10	4	1	2	7
90	900					-6	5	3	-1	5
92	920					-9	4	-1	1	8
94	940					-5	4	5	2	9
96	960					-9	8	7	4	12
98	980					-5	10	8	5	13
100	1000					0	9	5	7	17
102	1020					0	8	8	9	15
104	1040					-4	3	6	5	14
106	1060					-5	7	4	5	13
108	1080					0	9	5	4	12
110	1100					1	10	8	7	14
112	1120					6	10	11	11	14
114	1140					9	12	12	12	14
116	1160					9	17	14	13	16
118	1180					14	15	15	16	16
120	1200					14	11	11	8	14
122	1220					17	20	16	12	15
124	1240					13	21	17	15	17
126	1260					19	15	18	18	18
128	1280					11	17	19	18	16
130	1300					10	13	20	13	22
132	1320					12	0	18	15	20
134	1340					7		15	14	20
136	1360					5		14	12	18
138	1380					0		13	12	17
140	1400							11	13	19
142	1420							7	10	16
144	1440							6	8	17
146	1460							2	6	14
148	1480							0	0	8
150	1500									6
102	1520									3
104	1540									

The second hydrograph; Unsteady flow conditions; Circle culvert; blockage 0%

Circle, Unsteady, second Hydrograph, 225 min 0% Blockage										
**Distance (cm)**	**Distance (mm)**	2 lit/s or 7.2 m3/h, **25 min**	5 lit/s or 18 m3/h, **50 min**	8 lit/s 28.8 m3/h, **75 min**	11 lit/s or 39.6 m3/h, **100 min**	14 lit/s 50.4 m3/h, **125 min**	11 lit/s or 39.6 m3/h, **150 min**	8 lit/s 28.8 m3/h, **175 min**	5 lit/s or 18 m3/h, **200 min**	2 lit/s or 7.2 m3/h, **225 min**
0	0	-9	-15	-17	-26	-36	-39	-42	-43	-42
2	20	-20	-37	-38	-46	-49	-50	-52	-52	-52
4	40	-27	-40	-53	-55	-63	-53	-56	-60	-53
6	60	-40	-44	-45	-64	-69	-56	-61	-62	-55
8	80	-42	-49	-63	-63	-71	-70	-65	-65	-62
10	100	-43	-57	-64	-72	-63	-67	-67	-65	-60
12	120	-35	-56	-66	-74	-66	-64	-65	-66	-62
14	140	-41	-60	-67	-75	-74	-63	-60	-61	-60
16	160	-31	-54	-57	-73	-70	-64	-61	-56	-60
18	180	-23	-58	-63	-72	-73	-62	-60	-60	-58
20	200	-31	-57	-56	-76	-73	-66	-65	-61	-60
22	220	-27	-57	-61	-79	-67	-64	-63	-64	-62
24	240	-23	-59	-64	-72	-68	-67	-63	-69	-66
26	260	-16	-59	-67	-73	-67	-66	-66	-69	-67
28	280	-12	-61	-60	-72	-82	-66	-70	-74	-69
30	300	-10	-55	-59	-71	-79	-71	-73	-77	-73
32	320	-7	-48	-57	-75	-79	-74	-75	-76	-72
34	340	-5	-47	-63	-70	-80	-71	-72	-79	-72
36	360	3	-45	-65	-64	-73	-75	-74	-75	-75
38	380	5	-52	-69	-66	-76	-77	-73	-75	-70
40	400	3	-44	-56	-70	-72	-71	-72	-73	-79
42	420	0	-48	-55	-67	-70	-72	-73	-76	-68
44	440		-43	-57	-72	-67	-70	-74	-73	-70
46	460		-42	-53	-71	-74	-75	-76	-73	-68
48	480		-37	-46	-63	-72	-67	-77	-72	-70
50	500		-37	-47	-63	-68	-66	-70	-70	-66
52	520		-37	-49	-53	-65	-63	-70	-70	-65
54	540		-27	-47	-57	-67	-65	-67	-68	-65
56	560		-27	-46	-53	-66	-62	-67	-65	-68
58	580		-26	-49	-46	-62	-58	-69	-57	-63
60	600		-22	-43	-45	-60	-53	-66	-57	-62
62	620		-25	-37	-41	-57	-50	-63	-54	-58
64	640		-23	-30	-47	-51	-47	-62	-55	-55
66	660		-16	-35	-43	-48	-52	-56	-47	-50
68	680		-11	-22	-45	-47	-50	-57	-45	-50
70	700		-13	-20	-40	-43	-44	-53	-42	-46
72	720		-10	-19	-35	-33	-40	-50	-36	-43
74	740		-5	-19	-34	-37	-36	-42	-40	-38
76	760		-4	-17	-30	-35	-37	-40	-35	-35
78	780		-5	-13	-33	-26	-27	-36	-31	-30
80	800		-6	-10	-29	-30	-30	-36	-25	-30
82	820		-2	-6	-24	-24	-23	-34	-23	-25
84	840		-2	-5	-22	-26	-17	-29	-20	-20
86	860		0	-3	-13	-22	-17	-24	-14	-18
88	880			0	-10	-15	-13	-16	-7	-15
90	900			4	-13	-12	-12	-7	-6	-13
92	920			5	-7	-8	-8	-10	-6	-7
94	940			15	-4	-10	-6	-3	5	-3
96	960			13	-2	-7	-3	3	4	-2
98	980			17	1	5	4	5	3	2
100	1000			13	3	-2	7	4	6	4
102	1020			12	8	-4	6	6	12	7
104	1040			15	8	0	9	8	14	9
106	1060			20	10	4	9	13	17	10
108	1080			25	12	15	12	15	19	14
110	1100			24	10	15	14	14	20	15
112	1120			26	20	14	15	16	26	18
114	1140			22	22	14	19	20	25	22
116	1160			24	26	20	25	23	28	24
118	1180			25	26	20	28	20	32	24
120	1200			24	27	17	27	25	34	28
122	1220			13	29	20	27	30	28	30
124	1240			4	27	23	29	30	34	35
126	1260			0	23	25	33	27	32	30
128	1280				27	27	35	31	32	30
130	1300				30	28	31	30	32	32
132	1320				24	32	32	34	33	35
134	1340				24	29	31	32	32	35
136	1360				25	28	33	33	34	35
138	1380				18	25	35	35	30	37
140	1400				16	24	34	34	26	34
142	1420				8	23	30	31	15	30
144	1440				0	17	27	28	10	28
146	1460					13	22	22		34
148	1480					0	17	16		30
150	1500						10	15		28
102	1520						1.3	7		22
104	1540									17
106	1560									10
108	1580									3
110	1600									0

The second hydrograph; Unsteady flow conditions; Circle culvert; blockage 15%

Circle, Unsteady, second Hydrograph, 225 min 15% Blockage

**Distance (cm)**	**Distance (mm)**	2 lit/s or 7.2 m3/h, **25 min**	5 lit/s or 18 m3/h, **50 min**	8 lit/s 28.8 m3/h, **75 min**	11 lit/s or 39.6 m3/h, **100 min**	14 lit/s 50.4 m3/h, **125 min**	11 lit/s or 39.6 m3/h, **150 min**	8 lit/s 28.8 m3/h, **175 min**	5 lit/s or 18 m3/h, **200 min**	2 lit/s or 7.2 m3/h, **225 min**
0	0	0	0	0	0	0	0	0	0	0
2	20	-21	-31	-81	-78	-80	-77	-85	-77	-82
4	40	-32	-40	-87	-85	-78	-85	-86	-86	-73
6	60	-41	-67	-93	-90	-80	-79	-82	-85	-72
8	80	-40	-70	-95	-93	-81	-80	-81	-80	-71
10	100	-42	-83	-106	-96	-79	-78	-82	-83	-76
12	120	-36	-85	-102	-100	-80	-81	-82	-79	-75
14	140	-44	-83	-98	-90	-86	-81	-81	-79	-73
16	160	-42	-84	-93	-102	-84	-82	-82	-80	-77
18	180	-44	-80	-95	-101	-80	-82	-83	-83	-78
20	200	-42	-75	-105	-104	-86	-83	-85	-86	-80
22	220	-40	-73	-100	-108	-90	-83	-85	-86	-78
24	240	-27	-67	-92	-101	-88	-83	-87	-87	-83
26	260	-24	-66	-98	-108	-91	-90	-88	-91	-85
28	280	-26	-69	-93	-106	-89	-88	-92	-92	-82
30	300	-28	-70	-91	-100	-93	-93	-94	-95	-86
32	320	-24	-68	-85	-102	-96	-94	-97	-96	-93
34	340	-22	-66	-88	-100	-97	-95	-98	-96	-91
36	360	-21	-66	-90	-102	-105	-93	-102	-101	-95
38	380	-16	-64	-90	-102	-102	-94	-102	-100	-94
40	400	-15	-57	-88	-103	-98	-102	-105	-102	-96
42	420	-9	-53	-88	-100	-100	-102	-106	-104	-102
44	440	-11	-50	-90	-100	-98	-105	-104	-103	-104
46	460	-10	-49	-89	-99	-99	-105	-106	-102	-100
48	480	-10	-45	-86	-100	-100	-102	-106	-105	-95
50	500	-8	-36	-90	-96	-96	-102	-104	-106	-97
52	520	-7	-33	-90	-95	-95	-105	-102	-104	-102
54	540	-6	-30	-86	-98	-98	-106	-101	-104	-97
56	560	-5	-28	-82	-97	-97	-104	-100	-104	-98
58	580	-5	-24	-78	-96	-96	-102	-93	-104	-100
60	600	-3	-26	-79	-102	-102	-102	-105	-102	-102
62	620	0	-18	-77	-97	-97	-100	-102	-102	-98
64	640		-23	-73	-99	-99	-96	-100	-104	-92
66	660		-9	-66	-98	-98	-95	-96	-104	-95
68	680		0	-60	-96	-96	-93	-97	-100	-88
70	700			-57	-90	-90	-94	-91	-99	-89
72	720			-56	-85	-85	-89	-89	-97	-80
74	740			-48	-86	-86	-86	-86	-94	-75
76	760			-47	-85	-85	-83	-84	-86	-72
78	780			-39	-85	-85	-81	-80	-87	-75
80	800			-34	-80	-80	-78	-82	-83	-70
82	820			-28	-80	-80	-75	-78	-81	-68
84	840			-13	-70	-70	-74	-76	-75	-67
86	860			-20	-73	-73	-73	-73	-73	-69
88	880			-13	-72	-72	-70	-68	-72	-61
90	900			-15	-75	-66	-66	-66	-66	-60
92	920			-13	-64	-65	-61	-62	-66	-57
94	940			-8	-62	-65	-53	-62	-63	-56
96	960			-4	-59	-55	-55	-60	-58	-53
98	980			0	-46	-56	-50	-52	-54	-51
100	1000				-49	-56	-43	-52	-52	-50
102	1020				-47	-56	-39	-53	-49	-43
104	1040				-46	-46	-33	-49	-49	-42
106	1060				-40	-43	-30	-43	-40	-35
108	1080				-32	-40	-26	-41	-39	-33
110	1100				-31	-33	-25	-38	-30	-28
112	1120				-27	-30	-20	-33	-28	-22
114	1140				-25	-27	-15	-30	-24	-22
116	1160				-23	-28	-13	-25	-18	-19
118	1180				-13	-23	-5	-20	-16	-15
120	1200				-7	-21	-3	-15	-13	-10
122	1220				0	-16	-2	-12	-10	-8
124	1240					-13	-1	-7	-7	-4
126	1260					-6	0	-2	-1	-2
128	1280					-5		-2	-2	1
130	1300					0		-1	-3	2
132	1320							0	-1	5
134	1340								0	7
136	1360									15
138	1380									17
140	1400									20

The second hydrograph; Unsteady flow conditions; Circle culvert; blockage 30%

Circle, Unsteady, second Hydrograph, 225 min 30% Blockage

**Distance (cm)**	**Distance (mm)**	2 lit/s or 7.2 m3/h, **25 min**	5 lit/s or 18 m3/h, **50 min**	8 lit/s 28.8 m3/h, **75 min**	11 lit/s or 39.6 m3/h, **100 min**	14 lit/s 50.4 m3/h, **125 min**	11 lit/s or 39.6 m3/h, **150 min**	8 lit/s 28.8 m3/h, **175 min**	5 lit/s or 18 m3/h, **200 min**	2 lit/s or 7.2 m3/h, **225 min**
0	0	-10	-25	-36	-42	-53	-57	-59	-62	-67
2	20	-23	-46	-62	-68	-70	-73	-75	-67	-74
4	40	-34	-51	-64	-74	-73	-76	-74	-72	-71
6	60	-40	-65	-73	-73	-74	-77	-73	-74	-72
8	80	-42	-65	-76	-78	-72	-78	-73	-74	-70
10	100	-46	-67	-76	-78	-73	-73	-72	-73	-70
12	120	-43	-68	-78	-73	-77	-77	-71	-75	-74
14	140	-40	-69	-79	-77	-80	-79	-75	-76	-72
16	160	-42	-76	-82	-75	-77	-80	-76	-76	-80
18	180	-46	-72	-92	-74	-84	-79	-80	-79	-87
20	200	-40	-75	-100	-77	-90	-81	-84	-80	-98
22	220	-38	-80	-105	-75	-94	-87	-90	-83	-109
24	240	-32	-81	-110	-77	-100	-94	-89	-87	-118
26	260	-35	-90	-115	-78	-107	-100	-98	-95	-128
28	280	-35	-77	-108	-83	-119	-108	-107	-108	-133
30	300	-33	-74	-97	-98	-127	-110	-116	-116	-134
32	320	-30	-75	-87	-107	-120	-118	-128	-125	-130
34	340	-29	-72	-85	-119	-109	-127	-132	-135	-111
36	360	-29	-70	-86	-110	-97	-130	-130	-130	-102
38	380	-30	-69	-77	-107	-88	-123	-117	-111	-95
40	400	-24	-64	-78	-91	-82	-111	-108	-106	-81
42	420	-20	-61	-78	-84	-81	-109	-92	-97	-75
44	440	-18	-59	-77	-76	-80	-97	-87	-87	-74
46	460	-20	-60	-74	-75	-76	-88	-80	-80	-74
48	480	-16	-57	-76	-75	-76	-73	-72	-76	-72
50	500	-15	-52	-77	-76	-80	-70	-70	-72	-67
52	520	-13	-47	-78	-73	-75	-75	-70	-77	-70
54	540	-12	-45	-72	-70	-73	-70	-70	-74	-65
56	560	-5	-41	-70	-68	-74	-71	-69	-74	-65
58	580	-5	-42	-69	-67	-70	-70	-68	-69	-64
60	600	-3	-42	-66	-62	-69	-67	-70	-67	-64
62	620	0	-44	-66	-67	-68	-64	-67	-66	-63
64	640	2	-34	-64	-63	-69	-67	-64	-65	-62
66	660	3	-33	-62	-59	-70	-63	-63	-63	-60
68	680	5	-27	-60	-56	-62	-60	-64	-59	-57
70	700	5	-29	-56	-55	-55	-57	-60	-61	-55
72	720	7	-25	-59	-51	-62	-56	-56	-57	-53
74	740	9	-11	-65	-50	-55	-50	-53	-52	-50
76	760	10	-7	-61	-50	-62	-50	-49	-49	-45
78	780	6	-4	-54	-49	-50	-47	-48	-47	-44
80	800	5	4	-55	-42	-48	-45	-46	-46	-42
82	820	3	6	-49	-36	-47	-43	-44	-42	-42
84	840	0	8	-49	-35	-49	-40	-42	-40	-40
86	860		10	-44	-37	-44	-37	-40	-38	-37
88	880		13	-37	-37	-39	-35	-37	-36	-35
90	900		10	-27	-39	-37	-35	-34	-31	-34
92	920		14	-25	-34	-34	-30	-30	-30	-30
94	940		17	-22	-30	-35	-24	-29	-26	-26
96	960		23	-17	-26	-27	-27	-24	-23	-24
98	980		20	-15	-23	-26	-20	-19	-22	-19
100	1000		22	-13	-16	-24	-17	-20	-16	-20
102	1020		20	-12	-13	-23	-13	-17	-12	-13
104	1040		24	-12	-16	-16	-12	-12	-10	-11
106	1060		26	-5	-12	-11	-10	-10	-7	-7
108	1080		30	-2	-5	-9	-5	-7	-5	-4
110	1100		30	-5	-3	-7	-3	-4	-2	-1
112	1120		29	3	4	-4	-1	-2	1	-1
114	1140		30	5	8	-3	6	0	1	2
116	1160		30	7	14	0	7	6	7	4
118	1180		29	13	16	5	11	9	13	8
120	1200		28	11	23	9		13	19	13
122	1220		25	13	27	11		17	23	17
124	1240		25	17	30	14		20		21
126	1260		20	20	32	16				23
128	1280		18	23		21				24
130	1300		16							28
132	1320		10							30
134	1340		7							32
136	1360		6							34
138	1380		0							36
140	1400									38
142	1420									40
144	1440									40
146	1460									45
148	1480									48

The first hydrograph; Steady flow conditions; Box culvert; blockages 0%, 15%, and 30%

Box, 360 min, 0% Blockage		
Distance (mm)	Distance (mm)	Scour depth (mm)
0	0	-28
2	20	-51
4	40	-54
6	60	-57
8	80	-60.5
10	100	-66.5
12	120	-66.5
14	140	-75
16	160	-76
18	180	-82
20	200	-82
22	220	-86
24	240	-88
26	260	-93
28	280	-90
30	300	-92
32	320	-91
34	340	-89
36	360	-86
38	380	-83
40	400	-77
42	420	-74
44	440	-69
46	460	-67
48	480	-66
50	500	-57
52	520	-56
54	540	-47
56	560	-46
58	580	-43
60	600	-36
62	620	-27
64	640	-26
66	660	-24
68	680	-19
70	700	-16
72	720	-13
74	740	-6
76	760	-3
78	780	2
80	800	7
82	820	15
84	840	16
86	860	18
88	880	19
90	900	23.5
92	920	25
94	940	29
96	960	32
98	980	34
100	1000	37
102	1020	36
104	1040	40
106	1060	43
108	1080	43
110	1100	44
112	1120	44
114	1140	47
116	1160	49
118	1180	43
120	1200	45
122	1220	47
124	1240	43
126	1260	41
128	1280	42
130	1300	37
132	1320	36
134	1340	33
136	1360	32
138	1380	27
140	1400	23
142	1420	22
144	1440	23
146	1460	21.1
148	1480	19
150	1500	17

Box, 360 min, 15% Blockage		
Distance (cm)	Distance (mm)	Scour depth (mm)
0	0	-29
2	20	-31
4	40	-35
6	60	-46
8	80	-56
10	100	-63
12	120	-70
14	140	-72
16	160	-78
18	180	-80
20	200	-86
22	220	-85
24	240	-80
26	260	-85
28	280	-90
30	300	-95
32	320	-100
34	340	-93
36	360	-85
38	380	-78
40	400	-72
42	420	-68
44	440	-71
46	460	-68
48	480	-65
50	500	-64
52	520	-62
54	540	-55
56	560	-51
58	580	-54
60	600	-45
62	620	-45
64	640	-42
66	660	-35
68	680	-31
70	700	-26
72	720	-27
74	740	-27
76	760	-20
78	780	-16
80	800	-10
82	820	-4
84	840	-1
86	860	8
88	880	11
90	900	17
92	920	19
94	940	21
96	960	23
98	980	28
100	1000	32
102	1020	33
104	1040	35
106	1060	39
108	1080	38
110	1100	42
112	1120	43
114	1140	45
116	1160	43
118	1180	43
120	1200	45
122	1220	40
124	1240	39
126	1260	37
128	1280	36
130	1300	34
132	1320	31
134	1340	25
136	1360	20
138	1380	14
140	1400	9
142	1420	0

Box, 360 min, 30% Blockage		
Distance (cm)	Distance (mm)	Scour depth (mm)
0	0	-33
2	20	-37
4	40	-43
6	60	-50
8	80	-61
10	100	-67
12	120	-72
14	140	-75
16	160	-82
18	180	-85
20	200	-80
22	220	-86
24	240	-83
26	260	-100
28	280	-94
30	300	-93
32	320	-90
34	340	-76
36	360	-70
38	380	-71
40	400	-57
42	420	-53
44	440	-60
46	460	-58
48	480	-56
50	500	-52
52	520	-45
54	540	-43
56	560	-35
58	580	-33
60	600	-39
62	620	-30
64	640	-25
66	660	-12
68	680	-5
70	700	-6
72	720	12
74	740	14
76	760	10
78	780	16
80	800	20
82	820	22
84	840	26
86	860	32
88	880	35
90	900	40
92	920	44
94	940	48
96	960	50
98	980	47
100	1000	43
102	1020	42
104	1040	32
106	1060	33
108	1080	27
110	1100	23
112	1120	20
114	1140	16
116	1160	13
118	1180	9
120	1200	15

The first hydrograph; Steady flow conditions; Circle culvert; blockages 0%, 15%, and 30%

Circle, 360 min, 0% Blockage

Distance (mm)	Distance (mm)	Scour depth (mm)
0	0	-39
2	20	-56
4	40	-62
6	60	-60
8	80	-56
10	100	-60
12	120	-62
14	140	-65
16	160	-66
18	180	-70
20	200	-72
22	220	-75
24	240	-80
26	260	-83
28	280	-90
30	300	-93
32	320	-102
34	340	-105
36	360	-106
38	380	-112
40	400	-114
42	420	-115
44	440	-122
46	460	-126
48	480	-127
50	500	-128
52	520	-130
54	540	-131
56	560	-130
58	580	-126
60	600	-124
62	620	-125
64	640	-121
66	660	-124
68	680	-125
70	700	-123
72	720	-123
74	740	-116
76	760	-116
78	780	-115
80	800	-112
82	820	-109
84	840	-106
86	860	-102
88	880	-99
90	900	-96
92	920	-94
94	940	-93
96	960	-90
98	980	-85
100	1000	-82
102	1020	-77
104	1040	-75
106	1060	-78
108	1080	-69
110	1100	-67
112	1120	-63
114	1140	-61
116	1160	-54
118	1180	-51
120	1200	-44
122	1220	-40
124	1240	-36
126	1260	-32
128	1280	-27
130	1300	-23
132	1320	-20
134	1340	-13
136	1360	-10
138	1380	-3
140	1400	0
142	1420	3
144	1440	8
146	1460	15
148	1480	20
150	1500	27

Circle, 360 min, 15% Blockage

Distance (mm)	Distance (mm)	Scour depth (mm)
0	0	-47
2	20	-68
4	40	-69
6	60	-73
8	80	-54
10	100	-54
12	120	-56
14	140	-58
16	160	-63
18	180	-67
20	200	-74
22	220	-82
24	240	-89
26	260	-94
28	280	-99
30	300	-106
32	320	-110
34	340	-113
36	360	-116
38	380	-123
40	400	-124
42	420	-126
44	440	-130
46	460	-132
48	480	-130
50	500	-135
52	520	-133
54	540	-131
56	560	-132
58	580	-134
60	600	-133
62	620	-130
64	640	-132
66	660	-132
68	680	-133
70	700	-129
72	720	-127
74	740	-123
76	760	-124
78	780	-117
80	800	-115
82	820	-113
84	840	-115
86	860	-112
88	880	-106
90	900	-108
92	920	-100
94	940	-98
96	960	-99
98	980	-93
100	1000	-90
102	1020	-90
104	1040	-84
106	1060	-77
108	1080	-77
110	1100	-75
112	1120	-72
114	1140	-68
116	1160	-61
118	1180	-56
120	1200	-52
122	1220	-46
124	1240	-45
126	1260	-33
128	1280	-38
130	1300	-30
132	1320	-23
134	1340	-22
136	1360	-18
138	1380	-13
140	1400	-10
142	1420	-6
144	1440	0

Circle, 360 min, 30% Blockage

Distance (mm)	Distance (mm)	Scour depth (mm)
10	100	-62
12	120	-82
14	140	-77
16	160	-66
18	180	-57
20	200	-49
22	220	-46
24	240	-58
26	260	-62
28	280	-70
30	300	-72
32	320	-77
34	340	-80
36	360	-82
38	380	-82
40	400	-84
42	420	-86
44	440	-100
46	460	-90
48	480	-83
50	500	-81
52	520	-77
54	540	-79
56	560	-79
58	580	-83
60	600	-79
62	620	-79
64	640	-77
66	660	-75
68	680	-71
70	700	-72
72	720	-70
74	740	-68
76	760	-67
78	780	-63
80	800	-68
82	820	-65
84	840	-64
86	860	-65
88	880	-60
90	900	-59
92	920	-57
94	940	-60
96	960	-60
98	980	-57
100	1000	-56
102	1020	-60
104	1040	-52
106	1060	-55
108	1080	-57
110	1100	-50
112	1120	-48
114	1140	-47
116	1160	-50
118	1180	-44
120	1200	-43
122	1220	-40
124	1240	-37
126	1260	-33
128	1280	-30
130	1300	-28
132	1320	-28
134	1340	-27
136	1360	-22
138	1380	-18
140	1400	-17
142	1420	-15
144	1440	-10
146	1460	-9
148	1480	-12
150	1500	-7

The second hydrograph; Steady flow conditions; Box culvert; blockages 0%, 15%, and 30%

Box, 225 min, 0% Blockage

Distance (mm)	Distance (mm)	Scour depth (mm)
0	0	-17
2	20	-26
4	40	-30.5
6	60	-34.5
8	80	-40
10	100	-44
12	120	-51
14	140	-56.5
16	160	-60
18	180	-66
20	200	-67.5
22	220	-75
24	240	-73.5
26	260	-75.5
28	280	-76
30	300	-75
32	320	-74
34	340	-76
36	360	-68.5
38	380	-71
40	400	-67.5
42	420	-69
44	440	-69
46	460	-61
48	480	-58
50	500	-54
52	520	-47.5
54	540	-45.5
56	560	-37.5
58	580	-35
60	600	-30
62	620	-26
64	640	-24
66	660	-28
68	680	-13.5
70	700	-11
72	720	-5
74	740	-2.5
76	760	2.5
78	780	8
80	800	11
82	820	13
84	840	15
86	860	18
88	880	16.5
90	900	20
92	920	22.5
94	940	25
96	960	25.5
98	980	27
100	1000	29.5
102	1020	30
104	1040	33.5
106	1060	35
108	1080	33
110	1100	34
112	1120	34
114	1140	33
116	1160	35
118	1180	35
120	1200	35
122	1220	33
124	1240	33
126	1260	32
128	1280	30
130	1300	26
132	1320	28
134	1340	26
136	1360	26
138	1380	25
140	1400	23.5
142	1420	22
144	1440	22.5
146	1460	19
148	1480	19

Box, 225 min, 15% Blockage

Distance (mm)	Distance (mm)	Scour depth (mm)
0	0	-16
2	20	-18
4	40	-29
6	60	-33
8	80	-43
10	100	-50
12	120	-51
14	140	-53
16	160	-60
18	180	-62
20	200	-60
22	220	-70
24	240	-75
26	260	-83
28	280	-88
30	300	-80
32	320	-70
34	340	-56
36	360	-53
38	380	-50
40	400	-45
42	420	-46
44	440	-36
46	460	-36
48	480	-26
50	500	-27
52	520	-22
54	540	-12
56	560	-10
58	580	-6
60	600	-5
62	620	1
64	640	4
66	660	4
68	680	11
70	700	14
72	720	15
74	740	24
76	760	20
78	780	30
80	800	29
82	820	30
84	840	34
86	860	28
88	880	29
90	900	27
92	920	26
94	940	23
96	960	19
98	980	19
100	1000	17
102	1020	18
104	1040	12
106	1060	15
108	1080	10
110	1100	7
112	1120	6
114	1140	5
116	1160	5
118	1180	4
120	1200	2
122	1220	0

Box, 225 min, 30% Blockage

Distance (mm)	Distance (mm)	Scour depth (mm)
0	0	-19
2	20	-25
4	40	-27
6	60	-24
8	80	-39
10	100	-45
12	120	-43
14	140	-49
16	160	-52
18	180	-55
20	200	-62
22	220	-70
24	240	-75
26	260	-80
28	280	-83
30	300	-95
32	320	-81
34	340	-75
36	360	-61
38	380	-60
40	400	-51
42	420	-50
44	440	-52
46	460	-52
48	480	-50
50	500	-46
52	520	-49
54	540	-46
56	560	-45
58	580	-42
60	600	-38
62	620	-36
64	640	-40
66	660	-30
68	680	-28
70	700	-26
72	720	-26
74	740	-23
76	760	-21
78	780	-16
80	800	-15
82	820	-17
84	840	-13
86	860	-12
88	880	-11
90	900	-9
92	920	-7
94	940	-5
96	960	-4
98	980	0
100	1000	-1
102	1020	3
104	1040	4
106	1060	5
108	1080	7
110	1100	10
112	1120	12
114	1140	15
116	1160	17
118	1180	19
120	1200	24
122	1220	25
124	1240	27
126	1260	30
128	1280	34
130	1300	34
132	1320	39
134	1340	40
136	1360	42
138	1380	45
140	1400	45
142	1420	47
144	1440	48
146	1460	49
148	1480	50
150	1500	52

The second hydrograph; Steady flow conditions; Circle culvert; blockages 0%, 15%, and 30%

Circle, 225 min, 0% Blockage		
Distance (mm)	Distance (mm)	Scour depth (mm)
0	0	-32
2	20	-45
4	40	-49
6	60	-55
8	80	-57
10	100	-56
12	120	-58
14	140	-59
16	160	-61
18	180	-64
20	200	-64
22	220	-70
24	240	-72
26	260	-72
28	280	-76
30	300	-76
32	320	-78
34	340	-82
36	360	-86
38	380	-89
40	400	-90
42	420	-94
44	440	-94
46	460	-90
48	480	-89
50	500	-90
52	520	-90
54	540	-91
56	560	-91
58	580	-88
60	600	-86
62	620	-86
64	640	-85
66	660	-85
68	680	-83
70	700	-86
72	720	-71
74	740	-77
76	760	-73
78	780	-71
80	800	-66
82	820	-65
84	840	-58
86	860	-56
88	880	-53
90	900	-50
92	920	-48
94	940	-33
96	960	-36
98	980	-34
100	1000	-30
102	1020	-27
104	1040	-27
106	1060	-20
108	1080	-17
110	1100	-16
112	1120	-9
114	1140	-7
116	1160	-2
118	1180	-1
120	1200	0
122	1220	6
124	1240	11
126	1260	9
128	1280	12
130	1300	18
132	1320	24
134	1340	30
136	1360	30
138	1380	35
140	1400	33
142	1420	37
144	1440	37
146	1460	40
148	1480	50
150	1500	45

Circle, 225 min, 15% Blockage		
Distance (mm)	Distance (mm)	Scour depth (mm)
0	0	-46
2	20	-67
4	40	-65
6	60	-61
8	80	-66
10	100	-70
12	120	-71
14	140	-75
16	160	-76
18	180	-80
20	200	-79
22	220	-84
24	240	-85
26	260	-89
28	280	-90
30	300	-92
32	320	-94
34	340	-94
36	360	-96
38	380	-110
40	400	-116
42	420	-119
44	440	-111
46	460	-112
48	480	-110
50	500	-112
52	520	-108
54	540	-106
56	560	-104
58	580	-102
60	600	-101
62	620	-101
64	640	-96
66	660	-92
68	680	-90
70	700	-88
72	720	-86
74	740	-85
76	760	-79
78	780	-75
80	800	-75
82	820	-66
84	840	-66
86	860	-60
88	880	-58
90	900	-55
92	920	-54
94	940	-49
96	960	-48
98	980	-44
100	1000	-38
102	1020	-34
104	1040	-28
106	1060	-28
108	1080	-20
110	1100	-17
112	1120	-13
114	1140	-10
116	1160	-3
118	1180	0
120	1200	7
122	1220	7
124	1240	13
126	1260	18
128	1280	24
130	1300	25
132	1320	27
134	1340	31
136	1360	33
138	1380	36
140	1400	40
142	1420	39

Circle, 225 min, 30% Blockage		
Distance (mm)	Distance (mm)	Scour depth (mm)
0	0	-52
2	20	-57
4	40	-58
6	60	-58
8	80	-55
10	100	-53
12	120	-54
14	140	-56
16	160	-60
18	180	-63
20	200	-65
22	220	-67
24	240	-68
26	260	-67
28	280	-67
30	300	-73
32	320	-75
34	340	-77
36	360	-80
38	380	-80
40	400	-82
42	420	-85
44	440	-87
46	460	-86
48	480	-78
50	500	-72
52	520	-69
54	540	-67
56	560	-66
58	580	-64
60	600	-62
62	620	-59
64	640	-54
66	660	-53
68	680	-53
70	700	-54
72	720	-51
74	740	-50
76	760	-50
78	780	-50
80	800	-48
82	820	-46
84	840	-44
86	860	-44
88	880	-42
90	900	-40
92	920	-40
94	940	-36
96	960	-34
98	980	-37
100	1000	-30
102	1020	-28
104	1040	-23
106	1060	-25
108	1080	-20
110	1100	-19
112	1120	-17
114	1140	-15
116	1160	-11
118	1180	-7
120	1200	-5
122	1220	0
124	1240	1
126	1260	3
128	1280	7
130	1300	10
132	1320	14
134	1340	19
136	1360	24
138	1380	30
140	1400	34
142	1420	40
